# African trypanosomiasis: Synthesis & SAR enabling novel drug discovery of ubiquinol mimics for trypanosome alternative oxidase

**DOI:** 10.1016/j.ejmech.2017.09.067

**Published:** 2017-12-01

**Authors:** Ryan A. West, Oran G. O'Doherty, Trevor Askwith, John Atack, Paul Beswick, Jamie Laverick, Michael Paradowski, Lewis E. Pennicott, Srinivasa P.S. Rao, Gareth Williams, Simon E. Ward

**Affiliations:** a Sussex Drug Discovery Centre, University of Sussex, Brighton, BN1 9QJ, UK; b Medicines Discovery Institute, Cardiff University, Park Place, Cardiff, CF10 3AT, UK; c Novartis Institutes for Tropical Diseases, 5300 Chiron Way, California, 94608-2916, USA

**Keywords:** Antiprotozoal agents, Oxidoreductase, Medicinal chemistry, Synthesis design

## Abstract

African trypanosomiasis is a parasitic disease affecting 5000 humans and millions of livestock animals in sub-Saharan Africa every year. Current treatments are limited, difficult to administer and often toxic causing long term injury or death in many patients. Trypanosome alternative oxidase is a parasite specific enzyme whose inhibition by the natural product ascofuranone (AF) has been shown to be curative in murine models. Until now synthetic methods to AF analogues have been limited, this has restricted both understanding of the key structural features required for binding and also how this chemotype could be developed to an effective therapeutic agent. The development of 3 amenable novel synthetic routes to ascofuranone-like compounds is described. The SAR generated around the AF chemotype is reported with correlation to the inhibition of *T. b. brucei* growth and corresponding selectivity in cytotoxic assessment in mammalian HepG2 cell lines. These methods allow access to greater synthetic diversification and have enabled the synthesis of compounds that have and will continue to facilitate further optimisation of the AF chemotype into a drug-like lead.

## Highlights

• Synthesis of ascofuranone like inhibitors of trypanosome alternative oxidase.
• Structure activity relationships of trypanosome alternative oxidase inhibitors.
• Correlation of trypanosome alternative oxidase inhibition and *T. b. brucei* growth.

## Graphical abstract

**Figure:**
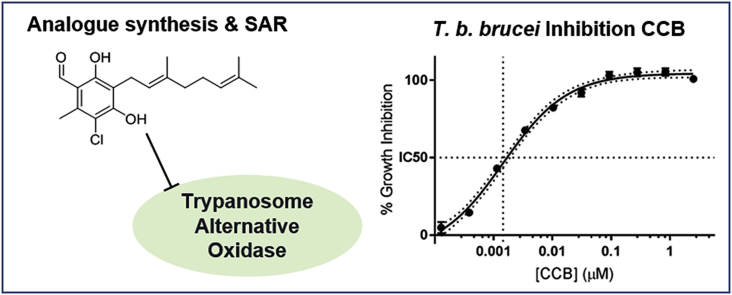

## 1 Introduction

African Trypanosomiasis is a parasitic protozoan infection in mammals spread by the tsetse fly (*Glossina*) [1]. It is exclusively found in 36 sub-Saharan African countries between 14°N and 20°S [2]. *Trypanosoma brucei gambiense* and *T. b. rhodesiense* cause the chronic western and acute eastern infections respectively, these two species have developed strategies to neutralize the immunity conferred by apolipoprotein A1, a trypanosomal lytic factor (TLF) in normal human sera [3], and evade host immune response by antigenic variation of their variant surface glycoprotein coat [4]. 60 million people are at risk of human African trypanosomiasis (HAT) with 5000 new cases reported annually [5], [6]. Stage 1 of the disease is haemolymphatic [7], subsequent penetration into the brain gives rise to stage 2 where meningoencephalitis and neuronal destruction produce a diverse range of symptoms including motor neuropathy, psychiatric disorders, sensory disturbances and the characteristic disruption of the diurnal cycle causing lethargy and insomnia that gives the disease its sleeping sickness title [8]. Without chemotherapeutic intervention the disease progresses to coma and death in almost all cases [9]. The high morbidity is evinced by the 1.79 million disability-adjusted life years (DALYs) calculated for HAT, over five times greater than that for the related disease leishmaniasis [10]. This infection is not limited to humans; animal African trypanosomiasis (AAT) is the single largest infection of cattle in Africa necessitating 35 million doses of trypanocidal agents, costing up to 140 million USD annually and impacting nutrition, livelihoods and development across sub-Saharan Africa. [11], [12] Different trypanosome species affect cattle, predominantly *T. vivax*, *T. evansi* and *T. congolense* [13]. AAT is less often fatal but its characteristic anaemia [14] severely impairs the health and productivity of cattle.

**Fig. 1:**
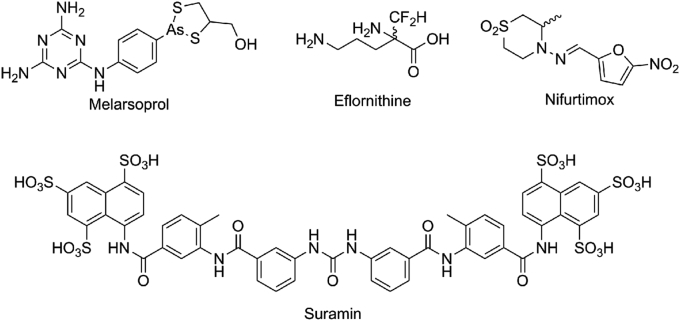
Current HAT treatments.

Current treatments for HAT are far from ideal: stage 1 treatments pentamidine and suramin (Fig. 1) only treat one subspecies (*T.b. gambiense* and *T.b. rhodesiense* respectively) and require intravenous/intramuscular (IV/IM) administration [15]. Similarly stage 2 is limited to nifurtimox-eflornithine combination therapy (NECT) and melarsoprol where NECT is ineffective in treating *T.b. rhodesiense* infection [15]. All current treatments are of limited utility due to significant toxicities; the organoarsenide melarsoprol even causes reactionary encephalitis in 10% of patients and death in 5%. [16], [17] The treatments all require clinicians for IV/IM injections [15], which is a major practical impediment for a diffuse population over a sizable portion of continental Africa. Drug resistance for melarsoprol and pentamidine has been increasingly observed [18] and has been linked to mutated aquaglyceroproteins both *in vitro* and in field isolates, required for trypanosomal uptake of the compounds [19].

With such poor therapeutics currently available, it is imperative that new, improved drug agents are discovered. An excellent target for drug development is trypanosome alternative oxidase (TAO). TAO is a ubiquinol dependent terminal oxidase required for the long slender blood stream trypanosomes aerobic glucose metabolism converting oxygen into water [20]. It is a 38 kDa cyanide-insensitive mitochondrial inner-leaf protein with a di-iron core which is key for enzymatic activity [21]. Many factors make it an exciting drug development target: 1) TAO is expressed in only parasitic organisms with no mammalian homologues [22]; 2) biochemical assays are well established [23]; 3) the crystal structure has been determined [24]; 4) inhibition of TAO has demonstrated clear trypanocidal action [25]; 5) existence of potent natural product inhibitors of TAO, in particular ascofuranone (AF) with Ki = 0.13 nM [26]; 6) demonstration of efficacy with ascofuranone *in vitro* and murine *in vivo* models [25], [27].

However, ascofuranone-like inhibitors have many undesirable qualities associated with their chemical structure. In particular, the electron rich aromatic ring, pendant aldehyde, phenols and lipophilic side chain contribute to the rapid observed clearance, low oral bioavailability and potential toxicity of this class. Furthermore, structure activity relationships (SAR) published around the geranyl tail clearly demonstrate that its effects are almost entirely due to non-specific lipophilic interactions.

Although TAO represents an attractive target for treating HAT, no real progress has been made in the development of potential drug agents from this chemical start point, despite the fact that AF has been known for over 20 years to be a potent inhibitor both *in vitro* and *in vivo.* [27], [28] One of the major bottlenecks to this progress has been lack of versatility of the available synthetic routes to these templates. There are only a handful of published synthetic methods, all suffering from linear sequence of steps resulting in poor overall yields [29]. [30] Additionally, our group has not been able to reproduce all of the chemistry reported to date. One key limitation of the current methodologies is that they rely on pre-formation of the penta-substituted aromatic head group, which is low-yielding and has limited potential for SAR expansion. The subsequent addition of the side chain is also capricious and low yielding. The reported deprotonation [31] with KOH followed by addition of geranyl bromide yielded only 5% product whilst other non-prenyl bromides preferably undergo O-alkylation. The selection of an appropriate protecting group for the bis-phenol **2** proved challenging as some silicon ethers or esters are labile in the presence of the *ortho*-aldehyde. The reported double SEM [32] protection of phenols in compound **3** with subsequent tail-chain addition *via* lithium halogen exchange was poor yielding, and subsequent removal of the SEM ethers proved impossible. Compound **2** does not undergo palladium-mediated coupling reactions, presumably due to the exposed phenols. Suzuki coupling is possible with methylated phenols but deprotection remains an impasse.

We report here a series of novel, simplified and improved synthetic routes to AF-like compounds. Two of the routes we have developed allow facile diversification of the head group that confers activity as well as alteration of the tail. We also report TAO inhibition data for the molecules prepared during these synthetic investigations. This will build upon the existing SAR platform around the active head group to help inform future development of AF-like TAO inhibitors for HAT.

## 2 Results and discussion

### 2.1 Synthetic chemistry

**Fig. 2:**
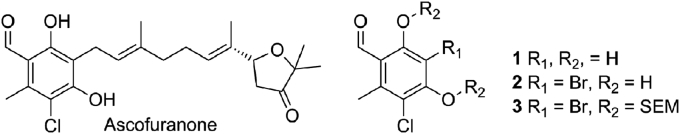
Ascofuranone and previous synthetic starting points.

**Scheme 1:**
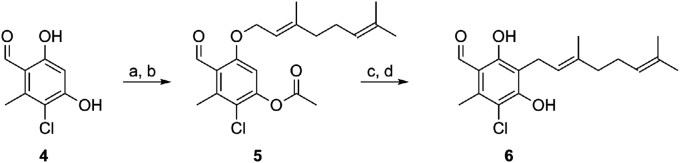
Lewis acid catalysed synthesis: a) NEt_3_, AcCl, CH_2_Cl_2_, 68%; b) geranyl bromide, K_2_CO_3_, KI, DMF, 50%; c) Florisil, toluene, 28**%**; d) NaOH, THF/MeOH, 97%.

Previous attempts to deprotect the bis-protected phenol ethers (Fig. 2) was found to only remove protecting groups *ortho* to the aldehyde. This was likely due to an enhanced bidentate chelation of Lewis acids available to this motif, this chelation could destabilise the etheric C-O bond and allow for its removal. To take advantage of this, Lewis-acid mediated cationic rearrangement of the prenyl chain was employed [33]. It was necessary to mask the *para* phenol as the acetate, to enable the rearrangement, as the unprotected *para* phenol substrate did not rearrange. Compound **5** was rearranged to give colletochlorin B (CCB-**6**) a proven trypanocide and potent TAO inhibitor (IC_50_–2 nM) in a yield which, although modest, was a significant improvement over past reports. This chemistry was subsequently employed to generate compounds in the following SAR investigation (see Scheme 1).

**Scheme 2:**
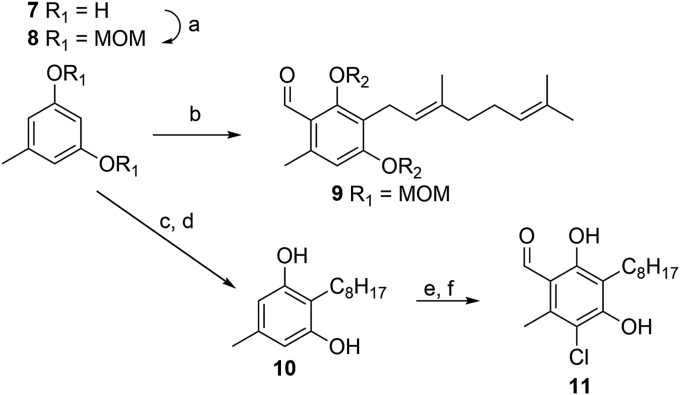
*Ortho*-lithiation syntheses: a) NaH, MOMCl, DMF, 93%; b) 1) *n*-BuLi, geranyl bromide, THF 2) *n*-BuLi, DMF, 48%; c) *n*-BuLi, bromooctane, THF, 0 °C, 95%; d) ethylene glycol, mw 160 °C, 98%; e) POCl_3_, DMF, 96%; f) SO_2_Cl_2_, CH_2_Cl_2_, quant.

Exploiting the reactivity of the phenolic functionality had proven successful and was explored further. When starting from orcinol, double protection of the phenols as MOM ethers was required to allow the reaction of alkyl bromides *via* lithiation and subsequent electrophilic aromatic substitution (exemplified by DMF in Scheme 2) in excellent yields. This alkylation was attempted with the geranyl chain, giving an overall 48% yield *via* sequential one-pot additions. However, removal of the protecting groups was again challenging yielding only 15% product. This method for addition of the sidechain takes advantage of the symmetrical starting material thus removing the regioselectivity issues from the subsequent additions.

**Scheme 3:**
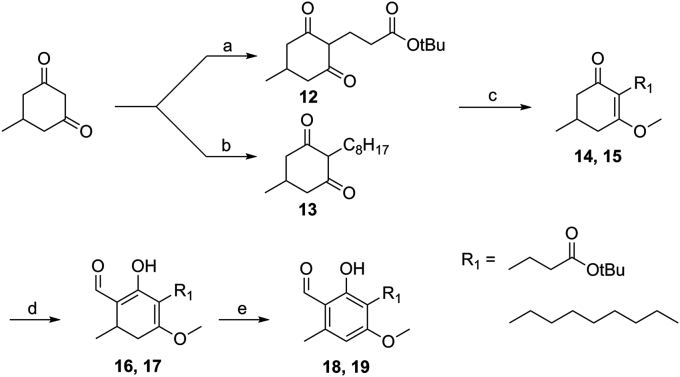
Diversifiable synthesis: a) NaH, ^t^Bu-acrylate, DMF, 80 °C, 95%, b) octanal, *l*-proline, NaBH_3_CN, EtOH, quant; c) SO_4_Me_2_, NaH, THF, 95%; d) LDA, methyl formate, THF, −78 °C, 64%; e) DDQ, CH_2_Cl_2_, 84%.

Further lithiation chemistry gave access to differentiated core structures through more flexible synthetic routes, however subsequent optimisation was required to improve both overall yields and increase structural diversity (Scheme 3). Starting from the diketone, various tails were installed, including those amenable to late stage diversification. Protection as the enol methyl ether **14** or **15** ensured clean formation of the desired enolate. This readily underwent substitution at the α-carbon. Oxidation by DDQ and subsequent de-protection afforded the AF-like analogues **18** and **19**.

A range of diverse analogues have been prepared with the chemistry described and are currently under biological investigation, data will be reported in future publications. These synthetic methods represent a significant improvement over previously reported methodologies and allow access to a range of diverse, novel AF-analogues. This work will facilitate the exploration of this chemotype to generate more drug like TAO inhibitors for future development.

### 2.2 Protein expression and purification

Recombinant TAO was expressed in Rosetta2^®^ *E. coli* by transformation of plasmid containing complementary DNA for the overexpression of His-tagged trypanosome alternative oxidase, obtained from Anthony Moore, University of Sussex. Subsequent culture in LB broth, containing 120 g/L sorbitol and 250 mM betaine, 50 μg/mL ampicillin and 35 μg/mL chloramphenicol, grown at 37 °C until OD_600_ 0.2 then grown at 18 °C until OD_600_ 0.6 was reached. The culture was induced with the addition of Isopropyl β-d-1-thiogalactopyranoside 500 μM final concentration and the cultures continued overnight at 18 °C. The *E. coli* pellet was isolated by centrifugation 10000*g* for 20 min at 4 °C (Beckman Coulter Avanti J26S, JLA9.1000 rotor). Lysis was carried out by re-suspension of the pellet in 50 mL of lysis buffer (50 mM Tris HCl, 20% (w/v) sucrose pH 7.4, 1 protease inhibitor cocktail tablet and 2 μL of Benzonase/DNAse^®^ Sigma) and sonication (Sonics Vibracell, 13 mm probe, 35% intensity, 180 s, 5 s pulses). Cell debris and unbroken cells were removed by centrifugation (Beckman Coulter Avanti J26S, JA 25.50 rotor, 8000 *g*, 10 min, 4 °C) to provide the supernatant as the crude fraction for further purification.

**Fig. 3:**
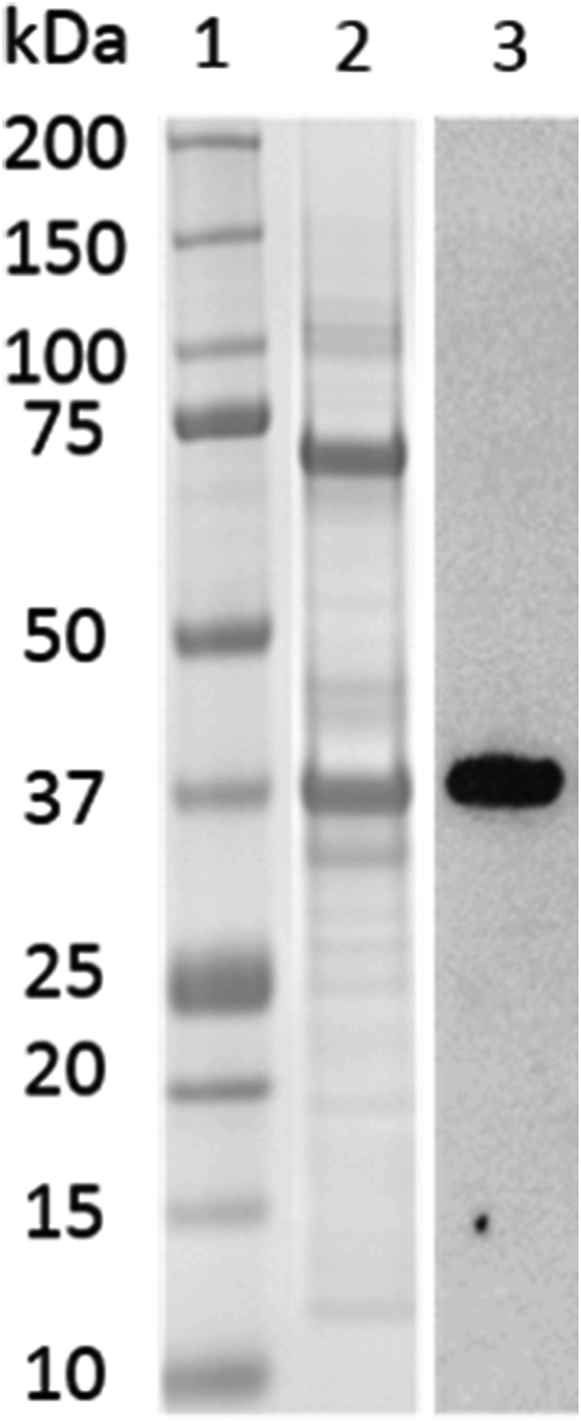
SDS-PAGE and Western analysis of rTAO with Quick Coomassie stain (Generon QC154201/008). 1 Molecular weight markers, 2 Concentrated purified rTAO protein, 3 WB 100 ng protein load. Polyclonal Ab from Agrisera (raised against plant AOX1/AOX2 (AS04 054) >70% homology to TAO).

**Fig. 4:**
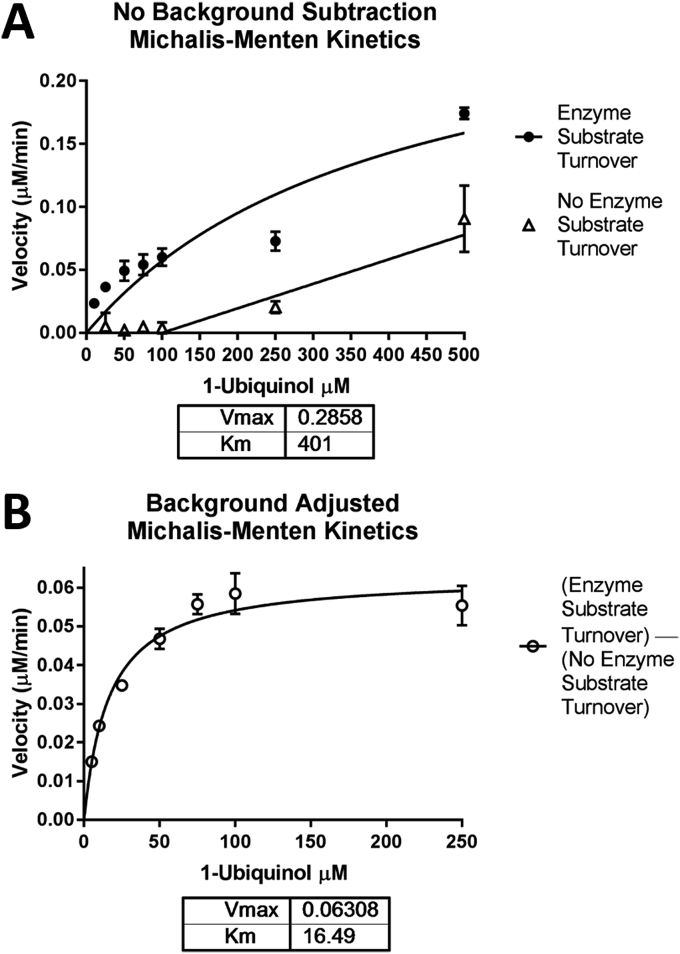
Michalis-Menten kinetics of rTAO. Data plotted is the mean of 3 independent experiments each with triplicate points for each condition. The error plotted is the SEM of the 3 independent experiments. The relative background turnover of substrate without enzyme was subtracted from each condition internally generated within each experiment in triplicate. 3 nM of purified rTAO was used. Graph generated in GraphPad Prism^®^.

Fractionation of the *E. coli* inner membranes was performed by isopycnic centrifugation. The crude supernatant was overlaid onto 45% sucrose solution (3 × 20 mL, 50 mM TrisHCl, 45% (w/v) sucrose, pH 7.4) and ultra-centrifuged (Beckman Coulter L-80, SW28, 141000 rcf max, 1 h, 4 °C) to isolate the *E. coli* inner membranes as the buoyant supernatant fraction. The supernatant from this was diluted 1:1 (50 mM Tris HCl, pH 7.4) to give a 15–20% sucrose solution reducing the density of the media and enabling the sedimentation of the inner membranes as a pellet by further ultracentrifugation (Beckman Coulter L-80, SW28, 141000 rcf max, 1 h, 4 °C). The inner membrane pellet was solubilised by re-suspension with octylglucopyranoside (OG) buffer (37 mL, 50 mM TrisHCl, 1.4% OG (w/v), 20% glycerol (v/v), 200 mM MgSO_4_, pH 7.4) followed by ultra-centrifugation (Beckman Coulter L-80, SW28, 141000 rcf max, 1 h, 4 °C) to remove non solubilised protein and debris. Purification of the solubilsed membrane fraction was performed by cobalt affinity chromatography (TALON^®^ resin). The column was equilibrated with buffer (50 mL, 20 mM Tris HCl, 1.4% OG, 20% glycerol, 100 mM MgSO_4_ pH 7.4). The resin was incubated with the solubilised supernatant for 30 min on a roller shaker at 4 °C. The flow through was collected and the resin was washed with buffer 1 (2 × 50 mL 20 mM TrisHCl, 20 mM imidazole, 0.042% (w/v) *n*-dodecyl-β-d-maltopyranoside (DDM), 20% (v/v) glycerol, 50 mM MgSO_4_, pH 7.4) and buffer 2 (1 × 50 mL 20 mM TrisHCl, 160 mM imidazole, 0.042% (w/v) *n*-dodecyl-β-d-maltopyranoside (DDM), 20% (v/v) glycerol, 50 mM MgSO_4_, pH 7.4). rTAO was then eluted with elution buffer (10 × 10 mL 20 mM TrisHCl, 200 mM imidazole, 0.042% (w/v) *n*-dodecyl-β-d-maltopyranoside (DDM), 20% (v/v) glycerol, 50 mM MgSO_4_, 60 mM NaCl, pH 7.4). Concentration of these fractions through a 10 kDa molecular weight cut off Amicon Ultra-15 filter unit provided TAO enzyme for use in absorbance inhibition assay, lane 2 Fig. 4. AOX confirmed in Western Blot analysis using Polyclonal Ab from Agrisera (raised against plant AOX1/AOX2 (AS04 054) >70% homology to TAO) (lane 3 Fig. 3).

Both bands observed at 37 kDa and 74 kDa in SDS-PAGE (lane 2) were confirmed to be rTAO by in-gel trypsin digest and analysis by protein mass spec (data not shown).

### 2.3 TAO activity assay

The assessment of enzymatic activity of purified rTAO was carried out by monitoring the production of 1-ubiquinone from the substrate 1-ubiquinol. This modified substrate was used over the endogenous substrate as it has a higher aqueous solubility and allows assessment of enzyme activity at higher substrate concentrations. The measurement of 1-ubiquinone production was carried out kinetically by absorbance readings at the substrates lambda max at 278 nm over time. Measurements were taken using assay buffer containing detergent C10E8 previously identified in assay optimisation experiments reported by Kido et al. [23] and 50 mM Tris buffer.

The Michalis-Menten kinetics of the purified enzyme was measured to determine the most suitable substrate concentration for use in screening activities. Interestingly we observed a Km of 16.5 μM which is notably lower than previous reports [23], [34]. Within our experiments we observed that kinetic measurements of product formation at high substrate concentrations (>250 μM) showed substantial rates of turnover. This was also observed in control experiments of substrate turnover without enzyme. If background subtractions of substrate turnover without enzyme were not performed calculations of Km showed to be in line with the previously reported figures for Km (**A** - Fig. 4). We therefore carried out background subtractions of turnover without enzyme at each concentration of substrate to generate Michalis-Menten Km calculations of enzyme alone (**B** - Fig. 4).

**Fig. 5:**
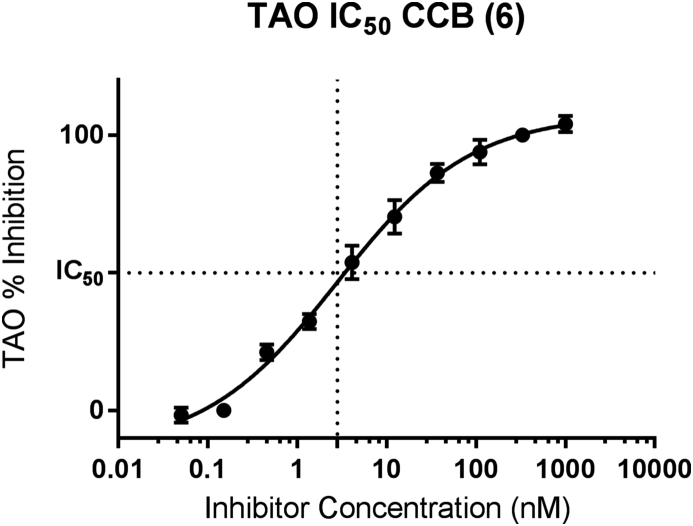
IC_50_ data for colletochlorin B from 4 parameter logistic regression of 10 point inhibitory concentration data. Data from 3 independent experiments reported concentrations run in duplicate per experiment, SEM plotted of experiments. 3 nM of purified rTAO was used.

**Fig. 6:**
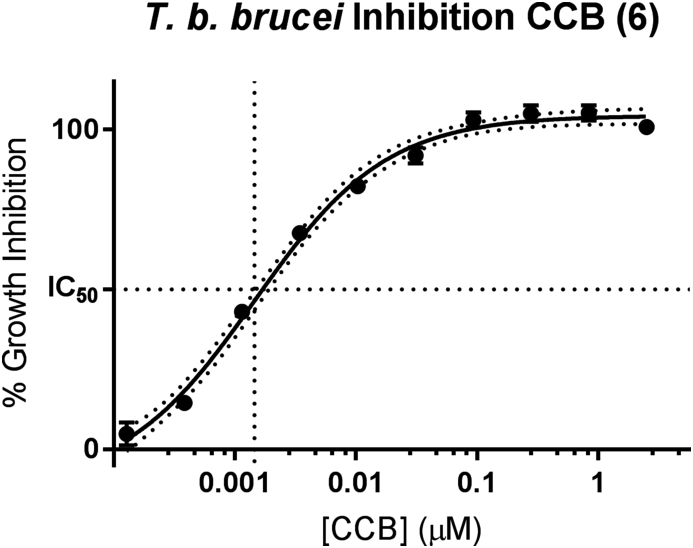
IC_50_ data for concentration of colletochlorin B required to inhibit 50% growth of *T. b. brucei* Lister427, following incubation with compound for 48 h. Growth inhibition was measured by quantifying ATP levels. 4 parameter logistic regression of 10 point inhibitory concentration data. Data from 3 independent experiments reported concentrations run in duplicate per experiment, SEM plotted of experiments.

In IC_50_ determination and in screening activities final concentrations of 3 nM of purified rTAO were used with 15 μM 1-ubiquinol for a sufficient window to assess the inhibitory activity of synthesised analogues. Using a substrate concentration close to the measured Km also enables the identification of competitive, non-competitive and uncompetitive types of inhibitors. We carried out ten-point IC_50_ determinations with purified rTAO protein to try to understand the action of these compounds. Example data for the natural product CCB – **6** is shown below (Fig. 5). With correlation to the *ex vivo* of inhibition of the growth of *T. b. brucei.* (Fig. 6).

**Fig. 7:**
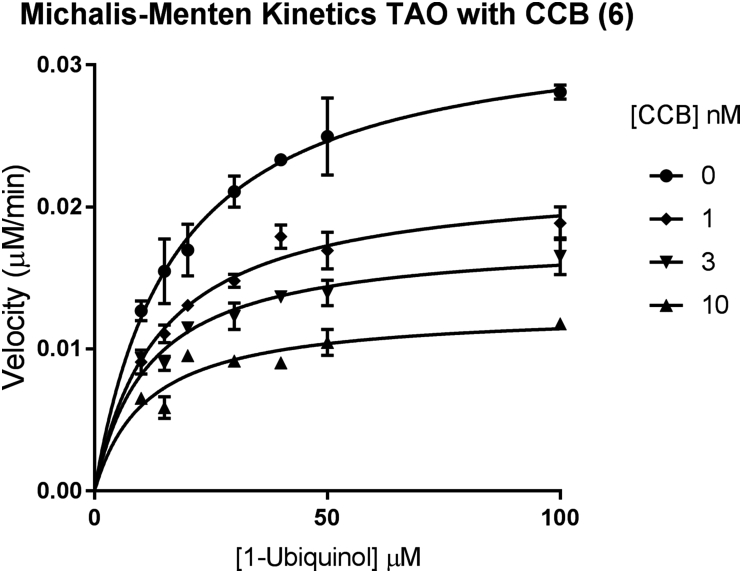
Michalis-Menten inhibitor kinetics of rTAO, in presence of increasing concentration of inhibitor CCB. Data plotted is the mean of 3 independent experiments each with triplicate points for each condition. The error plotted is the SEM of the 3 independent experiments. The relative background turnover of substrate without enzyme was subtracted from each condition internally generated within each experiment in triplicate. 2 nM of purified rTAO was used. Graph generated in GraphPad Prism^®^.

To understand the mechanism of TAO inhibition by colletochlorin B, more thorough experiments were performed, measuring the velocity of 1-ubiquinol turn over with increasing concentrations of colletochlorin B at different concentrations of substrate (Fig. 7). Interestingly this data suggests a mixed type non-competitive inhibition profile with regard to 1-ubiquinol substrate, that is in accordance with previous reports by Kido et al. [23] The apparent affinity of 1-ubiquinol is not affected by increasing concentrations of inhibitor as the calculated Km remains similar with increasing inhibitor concentration. Furthermore the V_max_ is reduced with increasing inhibitor concentration, which together with the Km observation are suggestive of non-competitive binding with regards to 1-ubiquinol.

### 2.4 Structure-activity relationships

At the outset of our research, there was not only a lack of robust synthetic routes to AF analogues but the basic SAR around the aromatic head group was under explored. Consequently, it was unclear which substituents were necessary for activity. Interestingly chlorination at R_2_ contributes a great deal to the inhibition of TAO. The des-chloro modifications to compounds **6** and **20–26** (Table 1) result in a reduction of 1–3 pIC_50_ units of potency against the enzyme, and diminishes selectivity for the growth of the trypanosome. Compound **23** shows 30 fold selectivity for inhibition of trypanosome growth compared to that of compound **6** that shows >500 fold selectivity over HepG2 cytotoxicity. Similarly the presence of a methyl group at R_3_ contributes significantly to the inhibition of TAO albeit to a lesser extent than that of chloro at R_2._ Compound **21** shows reduced inhibition of TAO that results in lower efficacy for trypanosome growth inhibition and consequently reduces the selectivity against the mammalian HepG2 cell to ∼60 fold.

**Table 1:**
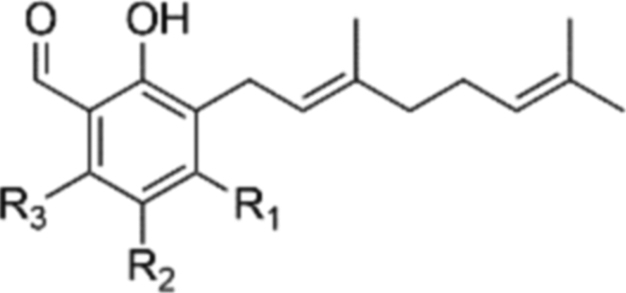
Summary of modifications to the aromatic head group.

#	R_1_	R_2_	R_3_	TAO inhibition (pIC_50_)^a^	*T.b.b.* Growth Inhibition (pIC_50_)^b^	HepG2 Cytotox (pCC_50_)^c^
**6**	OH	Cl	Me	8.4 ± 0.3	8.5 ± 0.1	5.1 ± 0.1
**20**	OAc	Cl	Me	6.5 ± 0.2	7.4 ± 0.7	4.8 ± 0.1
**21**	OH	Cl	H	7.0 ± 0.2	6.6 ± 0.1	<4.8
**22**	OAc	Cl	H	6.3 ± 0.1	6.2 ± 0.1	4.6 ± 0.1
**23**	OH	H	Me	5.5 ± 0.2	5.8 ± 0.1	4.4 ± 0.1
**24**	OAc	H	Me	5.4 ± 0.1	–	–
**25**	OH	H	H	5.4 ± 0.2	–	–
**26**	OAc	H	H	5.5 ± 0.1	–	–
**27**	H	Cl	H	5.4 ± 0.1	–	–
**28**	OH	H	OH	5.5 ± 0.1	–	–

a Negative log concentration and standard deviation of compounds required for 50% inhibition of trypanosome alternative oxidase.

b Negative log concentration and standard deviation of compounds required for 50% growth inhibition of *T. b. brucei* Lister427.

c Negative log concentration and standard deviation of compounds required for 50% growth inhibition of HepG2 cell line.

**Fig. 8:**
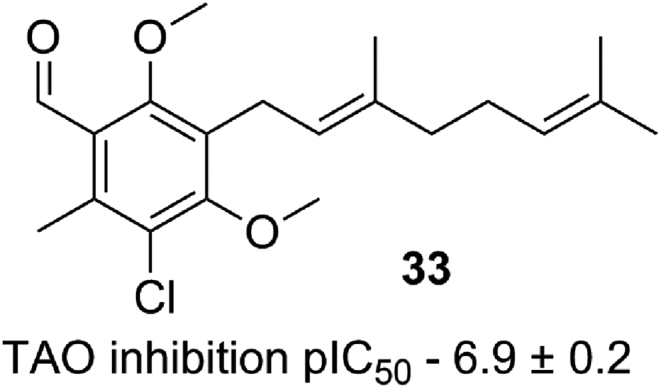
Dimethoxy analogue.

Previously published work had shown that electron withdrawing groups were preferred at R_4_ [26]. Our results confirm this with the exception of compound **30** where R_4_ is methyl. Direct comparison to compound **20** shows an order loss in potency suggesting that it is not only electron withdrawing groups could be tolerated in this position. Interestingly replacement of the benzaldehyde with nitrile **29** and **30** appeared to be a well-tolerated modification showing similar inhibition against TAO, however a drop in efficacy was observed against the trypanosome (see Table 2). One further divergence from the reported pharmacophore is compound **33** where the hydrogen bond donor of the phenol of C_5_ has been removed whilst retaining a modest inhibition of TAO, suggesting that the interaction with the enzyme at this position may not be *via* an essential hydrogen bond (see Fig. 8). This is supported by inhibition data of compound **35** where the point of attachment of the lipophilic tail was explored. The compound retains activity against TAO supporting the hypothesis that the necessity for a hydrogen bond donor at R_5_ is not a requirement for TAO inhibition. Ether-linked tails could thus reasonably represent a group of underexplored TAO inhibitors that are more synthetically amenable (see Table 3).

**Table 2:**
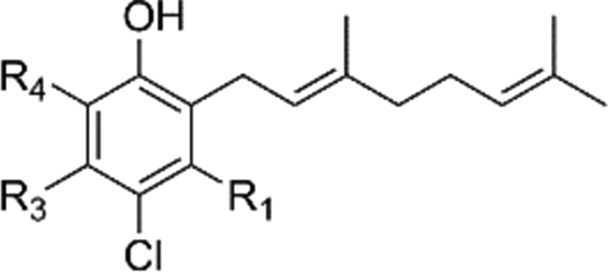
Summary of modifications to the aldehyde.

#	R_1_	R_3_	R_4_	TAO inhibition (pIC_50_)^a^	*T.b.b.* Growth inhibition (pIC_50_)^b^	HepG2 Cytotox (pCC_50_)^c^
**29**	OH	Me	CN	8.4 ± 0.2	6.4 ± 0.1	4.9 ± 0.1
**6**	OH	Me	C=O	8.5 ± 0.3	8.4 ± 0.1	5.1 ± 0.1
**20**	OAc	Me	C=O	6.5 ± 0.2	7.4 ± 0.7	4.8 ± 0.1
**30**	OAc	Me	Me	5.7 ± 0.1	–	–
**21**	OH	H	C=O	7.0 ± 0.2	6.6 ± 0.1	<4.8
**31**	OH	H	CN	7.6 ± 0.3	5.2 ± 0.1	4.4 ± 0.1
**32**	OH	H	C=NOH	6.1 ± 0.3	5.7 ± 0.1	5.0 ± 0.1

a Negative log concentration and standard deviation of compounds required for 50% inhibition of trypanosome alternative oxidase.

b Negative log concentration and standard deviation of compounds required for 50% growth inhibition of *T. b. brucei* Lister427.

c Negative log concentration and standard deviation of compounds required for 50% growth inhibition of HepG2 cell line.

**Table 3:**
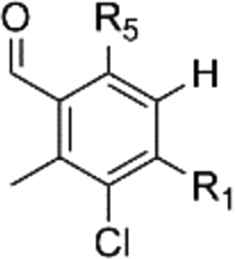
Alkylation of phenols.

#	R_1_	R_5_	TAO inhibition (pIC_50_)^a^	*T.b.b.* Growth inhibition (pIC_50_)^b^	HepG2 Cytotox (pCC_50_)^c^
**34**	*O*-Geranyl	OH	6.0 ± 0.1	5.5 ± 0.1	4.7 ± 0.1
**35**	OH	*O*-Geranyl	6.6 ± 0.2	4.9 ± 0.1	5.0 ± 0.1

a Negative log concentration and standard deviation of compounds required for 50% inhibition of trypanosome alternative oxidase.

b Negative log concentration and standard deviation of compounds required for 50% growth inhibition of *T. b. brucei* Lister427.

c Negative log concentration and standard deviation of compounds required for 50% growth inhibition of HepG2 cell line.

In general a reasonable correlation was observed between the inhibition of TAO and the inhibition of growth of the parasite. The OAc masked phenol in compound **20** showed higher inhibitory activity against *T. b. brucei* than expected. This higher than expected potency could be from the cleavage of the acetate to the free phenol compound **6** under the conditions of the assay. The majority of the compounds synthesised showed greater than 20 fold selectivity for *T. b. brucei* growth inhibition over mammalian HepG2 cytotoxicity. In general compounds that showed more potent inhibition of TAO showed better a better selectivity index over mammalian HepG2 cells (see Table 3).

## 3 Conclusions

Despite current efforts, HAT remains a considerable threat to the health and well-being of those infected. Currently available treatments are far from safe with increasing incidences of drug resistance being reported. Ascofuranone was described as a nanomolar inhibitor of TAO in the late 1990s [27], however exploitation of this opportunity has been severely limited due to the availability of robust synthetic routes to these molecules. With the potential to assist future drug development of the ascofuranone chemotype we present 3 novel and versatile synthetic approaches, together with the SAR findings developed during our synthetic exploration. These routes offer significantly improved yields over previous methods and importantly, allow late stage diversification of the crucial aromatic head group that is responsible for both the affinity for the enzyme and the undesirable, non-drug-like functionality. The SAR presented builds on that previously published [26], demonstrating that the aromatic pharmacophore is more amenable to alteration than previously reported. These synthetic and SAR findings provide the foundations for onward exploitation of this chemotype towards novel treatments for HAT *via* inhibition of TAO and our onward investigation further aims to develop CNS drug-like leads from these challenging chemical starting points.

## 4 Experimental section

Experimental Details. ChemAxon Calculator Plugins were used for structure property prediction and calculation (clogP), Marvin 15.5.4, 2015, ChemAxon (http://www.chemaxon.com). All commercial reagents were purchased from Sigma-Aldrich, Alfa Aesar, Apollo Scientific, Fluorochem or Tokyo Chemical Industry and of the highest available purity. Unless otherwise stated, chemicals were used as supplied without further purification. Anhydrous solvents were purchased from Acros (AcroSeal™) or Sigma-Aldrich (SureSeal™) and were stored under nitrogen. 40–60 petrol ether refers to the fraction with a boiling point between 40 °C and 60 °C. Anhydrous solvents and reagents were used as purchased. Thin layer chromatography (TLC) was carried out using glass plates pre-coated with Merck silica gel 60 F254. Melting points were determined using an OptiMelt apparatus and are uncorrected. Proton nuclear magnetic resonance spectra were recorded at 500 MHz on a Varian VNMRS 500 MHz spectrometer (at 30 °C), using residual isotopic solvent (CHCl_3_, *δ*_H_ = 7.27 ppm, DMSO *δ*_H_ = 2.50 ppm, MeOH *δ*_H_ = 3.31 ppm) as an internal reference. Chemical shifts are quoted in parts per million (ppm). Coupling constants (J) are recorded in Hertz (Hz). Carbon nuclear magnetic resonance spectra were recorded at 125 MHz on a Varian 500 MHz spectrometer and are proton decoupled, using residual isotopic solvent (CHCl_3_, *δ*_C_ = 77.00 ppm, DMSO *δ*_C_ = 39.52 ppm, MeOH *δ*_C_ = 49.00 ppm) as an internal reference. Proton and carbon spectra assignments are supported by DEPT editing. Chemical shifts (*δ*_C_) are quoted in ppm. High resolution mass spectrometry (HRMS) data (ESI) was recorded on Bruker Daltonics, Apex III, ESI source: Apollo ESI with methanol as spray solvent. Only molecular ions, fractions from molecular ions and other major peaks are reported as mass/charge (*m*/*z*) ratios. LCMS data was recorded on a Waters 2695 HPLC using a Waters 2487 UV detector and a Thermo LCQ ESI-MS. Samples were eluted through a Phenomenex Lunar 3 μm C18 50 mm × 4.6 mm column, using water and acetonitrile acidified by 0.1% formic acid at 1 ml/min and detected at 254 nm. The gradient employed was a 7 min method 30–90% MeCN over a 5 min gradient, held at 90% MeCN for 1 min, then re-equilibrated to 30% MeCN over 1 min. All experiments were carried out under an inert atmosphere of N_2_ unless otherwise stated.

**Absorbance Assay.** 1-Ubiquinol turnover was measured by recording the increase in absorbance at 278 nm (Greiner 96-well UV star flat bottom plates with BMG PHERAstar FS photo spectrometer) to monitor the increase of 1-Ubiquinone concentration kinetically over 6 min with purified rTAO (3 nM), The Michalis-Menten derived Km of 1-Ubiquinol showed to be 16.5 μM, kinetic measurements at high (>250 μM) concentrations of substrate showed higher rates of enzyme product formation, this was also observed with high substrate concentrations without enzyme. It was therefore necessary to carry out a background subtraction of substrate turnover without enzyme to generate accurate Michalis-Menten Km calculations. A final concentration of 15 μM 1-Ubiquinol was used under the following conditions: 50 mM Tris-HCl; 0.05% (w/v) C10E8; pH 7.4; 25 °C). The sigmoidal curve of the inhibition was observed by 10-point 3 fold serial dilution of test compounds to generate IC_50_ values.

**Growth inhibition assays.** Bloodstream form *Trypanosoma brucei brucei* Lister 427 parasites were continuously passaged in HMI-9 medium formulated from IMDM medium (Invitrogen), 10% heat-inactivated fetal bovine serum, 10% Serum Plus medium supplement (SAFC Biosciences), 1 mM hypoxanthine (Sigma-Aldrich), 50 μM bathocuproine disulfonic acid (Sigma-Aldrich), 1.5 mM cysteine (Sigma-Aldrich), 1 mM pyruvic acid (Sigma-Aldrich), 39 μg/mL thymidine (Sigma-Aldrich), and 14 μL/L beta-mercapthoethanol (Sigma-Aldrich); all concentrations of added components refer to that in complete HMI-9 medium. The parasites were cultured in 10 mL of HMI-9 medium in T75 CELL-STAR tissue culture flasks at 37 °C/5% CO_2_.

To determine growth inhibitory potency of compounds against *T. b. brucei* bloodstream form parasites, 200 nL of 10-point, 3 fold serially diluted compounds in DMSO were transferred to the wells of white, solid bottom 384-well plates (Greiner Bio-One) by either Echo 555 acoustic liquid handling system or Mosquito. Then, 10^4^ of *T. b. brucei* parasites in 40 μL of HMI-9 medium were added to each well, and the plates were incubated for 48 h at 37 °C in 5% CO_2_ incubators. Parasite numbers in individual plate wells were determined through quantification of intracellular ATP amount. The CellTiter-Glo luminescent cell viability reagent (Promega) was added to plate wells, and ATP-dependent luminescence signal was measured on Tecan M1000 plate Reader after 30 min incubation. Suramin an *anti*-trypanosomal drug was used positive control and DMSO was used as negative control. pIC_50_ values were calculated using Graph Pad Prism software by plotting the luminescence values in sigmoidal dose response curves. Suramin was used as a positive control in screening. (pIC_50_–6.7 ± 0.1).

**Hep-G2 Cytotoxicity Assay.** Human hepatocellular carcinoma (HepG2) cells were obtained from ATCC and grown in RPMI media. 25 μL of 1.6 × 10^4^ cells/mL were dispensed into sterile 384 well Griener clear plates and incubated at 37 °C in 5% CO_2_ incubator for 24 h. Once the cells adhered, 125 nL of 10-point, 3 fold serially diluted compounds in DMSO were transferred on to cells. After incubating for additional 96 h at 37 °C in 5% CO_2_ incubator, cells were added with CCK-8 reagent to each well. Plates were further incubated for 3 h followed by absorbance reading at 450 nM using Envision reader. Absorbance values were used for determination of cytotoxic concentration (pCC_50_) required to inhibit 50% growth. Purmycin was used as positive control in screening (pIC_50_–6.3 ± 0.1).

### 4.1 Synthesis

#### 4.1.1 2-Chloro-5-{[(2E)-3,7-dimethylocta-2,6-dien-1-yl]oxy}-4-formyl-3-methylphenyl acetate **(5)**

To a suspension of 3-chloro-4,6-dihydroxy-2-methylbenzaldehyde (**4**) [35] [36] (11.8 g, 63.2 mmol) in DCM (300 mL) was added trimethylamine (8.8 mL, 63.2 mmol) at room temperature. This mixture was stirred for 15 min during which time the suspension had dissolved. Acetyl chloride (4.5 mL, 63.3 mmol) was added to the reaction mixture dropwise at room temperature over 20 min and this was stirred for a further 5 h. Deionised water (200 mL) was added and the phases were separated. The aqueous layer was extracted with ethyl acetate (3 × 100 mL) and the combined organic extracts were dried over magnesium sulfate, filtered and concentrated under reduced pressure. Purification 2 times by flash column chromatography (100 g silica), eluting with a gradient of petrol ether: ethyl acetate (95: 5) to (90: 10), gave 2-chloro-4-formyl-5-hydroxy-3-methylphenyl acetate as an off-white solid (9.9 g, 68%): R_f_ 0.46 (10% ethyl acetate/90% 40–60 petrol ether); ^1^H NMR (500 MHz, Chloroform-*d*) *δ* 12.16 (s, 1H), 10.31 (s, 1H), 6.70 (s, 1H), 2.68 (s, 3H), 2.37 (s, 3H).

To a solution of 2-chloro-4-formyl-5-hydroxy-3-methylphenyl acetate (1.30 g, 5.7 mmol) and geranyl bromide (1.35 mL, 6.8 mmol) in dimethylformamide (10 mL) was added potassium iodide (85 mg, 0.6 mmol) followed by potassium carbonate (2.35 g, 17.1 mmol). The reaction mixture was stirred at room temperature for 16 h before being diluted with deionised water (50 mL). This mixture was extracted with diethyl ether (3 × 30 mL) and the combined organic extracts were dried over magnesium sulfate, filtered and concentrated under reduced pressure. Purification by flash column chromatography (25 g silica), eluting with a gradient of petrol ether: ethyl acetate (100: 0) to (90: 10), gave the title compound as a yellow oil (1.04 g, 50%) and 4-chloro-5-{[(2E)-3,7-dimethylocta-2,6-dien-1-yl]oxy}-2-formyl-3-methylphenyl acetate (710 mg, 41%).

#### 4.1.2 2-Chloro-5-{[(2E)-3,7-dimethylocta-2,6-dien-1-yl]oxy}-4-formyl-3-methylphenyl acetate **(5)**

R_f_ 0.46 (10% ethyl acetate/90% 40–60 petrol ether) ^1^H NMR (500 MHz, Chloroform-*d*) *δ* 10.56 (s, 1H), 6.71 (s, 1H), 5.51–5.40 (m, 1H), 5.13–5.05 (m, 1H), 4.60 (d, *J* = 6.6 Hz, 2H), 2.67 (s, 3H), 2.38 (s, 3H), 2.22–1.97 (m, 4H), 1.73 (s, 3H), 1.69 (s, 3H), 1.62 (s, 3H). LCMS (ESI) retention time 6.64 min, *m/z* 365.77/367.26.

#### 4.1.3 4-Chloro-5-{[(2E)-3,7-dimethylocta-2,6-dien-1-yl]oxy}-2-formyl-3-methylphenyl acetate (5)

R_f_ 0.28 (10% ethyl acetate/90% 40–60 petrol ether) ^1^H NMR (500 MHz, Chloroform-*d*) *δ* 10.26 (s, 1H), 6.58 (s, 1H), 5.57–5.41 (m, 1H), 5.09 (t, *J* = 6.1 Hz, 1H), 4.67 (d, *J* = 6.5 Hz, 2H), 2.72 (s, 3H), 2.38 (s, 3H), 2.18–2.07 (m, 4H), 1.76 (s, 3H), 1.69 (s, 3H), 1.62 (s, 3H). LCMS (ESI) retention time 6.64 min, *m/z* 365.59/367.66.

#### 4.1.4 3-Chloro-5-[(2E)-3,7-dimethylocta-2,6-dien-1-yl]-4,6-dihydroxy-2-methylbenzaldehyde **(6)**

(colletochlorin B) To a solution of 2-chloro-5-{[(2E)-3,7-dimethylocta-2,6-dien-1-yl]oxy}-4-formyl-3-methylphenyl acetate (**5**) (757 mg, 2.1 mmol) in toluene (7.5 mL) was added Fluorosil (100–200 mesh, 2.3 g). The reaction mixture was heated at reflux for 3 h before being allowed to cool to room temperature and being filtered. The Fluorosil was washed with ethyl acetate (3 × 15 mL) and the combined filtrate was concentrated under reduced pressure. Purification by flash column chromatography (25 g silica), eluting with a gradient of petrol ether: ethyl acetate (100: 0) to (95: 5), gave 2-chloro-6-[(2E)-3,7-dimethylocta-2,6-dien-1-yl]-4-formyl-5-hydroxy-3-methylphenyl acetate as a colourless oil (212 mg, 28%): R_f_ 0.67 (5% ethyl acetate/95% 40–60 petrol ether); ^1^H NMR (500 MHz, Chloroform-*d*) *δ* 12.53 (s, 1H), 10.31 (s, 1H), 5.21–4.96 (m, 2H), 3.35–3.25 (m, 2H), 2.66 (s, 3H), 2.38 (s, 3H), 2.09–2.01 (m, 2H), 2.01–1.93 (m, 2H), 1.75 (s, 3H), 1.66 (s, 3H), 1.59 (s, 2H).

To a solution of 2-chloro-6-[(2E)-3,7-dimethylocta-2,6-dien-1-yl]-4-formyl-5-hydroxy-3-methylphenyl acetate (110 mg, 0.3 mmol) in THF (4 mL) and MeOH (1 mL) was added 1 M aqueous sodium hydroxide (1.5 mL, 1.5 mmol). The reaction mixture was stirred at room temperature for 3 h during which time the reaction mixture turned deep purple. The reaction mixture was acidified to pH 1 with 1 M aqueous HCl and the reaction colour faded to yellow. This mixture was extracted with ethyl acetate (3 × 10 mL) and the combined organic extracts were dried over magnesium sulfate, filtered and concentrated under reduced pressure. Purification by flash column chromatography (10 g silica), eluting with a gradient of petrol ether: ethyl acetate (100: 0) to (95: 5), gave the title compound as an off-white solid (93 mg, 97%): R_f_ 0.48 (5% ethyl acetate/95% 40–60 petrol ether); ^1^H NMR (500 MHz, Chloroform-*d*) *δ* 12.70 (s, 1H), 10.16 (s, 1H), 6.42 (s, 1H), 5.29–5.17 (m, 1H), 5.09–5.05 (m, 1H), 3.42 (d, *J* = 7.1 Hz, 2H), 2.61 (s, 3H), 2.13–2.03 (m, 2H), 2.03–1.97 (m, 2H), 1.80 (s, 3H), 1.66 (d, *J* = 1.4 Hz, 3H), 1.59 (s, 3H).

#### 4.1.5 1,3-Bis(methoxymethoxy)-5-methylbenzene **(8)**

60% Sodium hydride in mineral oil (742 mg, 18.5 mmol) was added in three portions to a stirred solution of orcinol (**7**) (1.00 g, 8.1 mmol) in DMF (20 mL) at 0 °C. This was stirred for 40 min and methyl chloromethoxy ether (1.35 mL, 17.7 mmol) added dropwise. The reaction was stirred for 16 h and then diluted with water (100 mL) and extracted with diethyl ether (3 × 100 mL). The combined ether fractions were washed with 1 M sodium hydroxide solution (50 mL), saturated brine solution (50 mL), dried over sodium sulfate, filtered and concentrated under reduced pressure. Purification by flash column chromatography (20 g silica), eluting with a gradient of 40–60 petrol ether: ethyl acetate (100: 0) to (95: 5), gave the title compound as a clear oil (1.59 g, 93%): ^1^H NMR (500 MHz, chloroform-d) *δ* 6.56 (1 H, s), 6.54 (2H, s), 5.15 (4 H, s), 3.49 (6 H, s), 2.31 (3 H, s); HRMS (ESI) calcd 212.1049 for C_11_H_16_O_4_ [M+H]+, found 212.1047 *m/z*.

#### 4.1.6 (E)-3-(3,7-Dimethylocta-2,6-dienyl)-2,4-bis(methoxymethoxy)-6-methylbenzaldehyde **(9)**

3-Bis(methoxymethoxy)-5-methylbenzene (**8**) (2.50 g, 11.8 mmol) was dissolved in dry tetrahydrofuran (90 mL) and cooled to 0 °C. 2.1 M *n*-butyl lithium (8.41 mL, 17.7 mmol) was added drop-wise and the reaction was left to stir for 1 h at 0 °C. Geranyl bromide (4.48 g, 20.6 mmol) was added to the reaction mixture in a drop-wise manner. This was stirred for 1 h and allowed to come to room temperature. The reaction was cooled to 0 °C again and 2.1 M *n*-butyl lithium (8.41 mL, 17.7 mmol) added followed by another 1 h stirring at 0 °C. Excess dry DMF was added and the reaction was allowed to come to room temperature over 1 h. The reaction was diluted with water (300 mL) and extracted with diethyl ether (3 × 200 mL). The combined organics were dried over sodium sulfate, filtered and concentrated under reduced pressure. Purification by flash column chromatography (50 g silica), eluting with a gradient of 40–60 petrol ether: ethyl acetate (100:0) to (95:5), gave the title compound as a yellow tinted clear oil (1.28 g, 31%): ^1^H NMR (500 MHz, chloroform-d) *δ* 10.38 (1 H, s), 6.74 (1 H, s), 5.24 (2 H, s), 5.17 (1 H, t, *J* = 6.6 Hz), 5.04 (2 H, s), 5.01 (1 H, t, *J* = 6.4 Hz), 3.57 (3 H, s), 3.46 (3 H, s), 3.36 (2 H, d, *J* = 6.7 Hz), 2.57 (3 H, s), 2.05 (2 H, m), 1.97 (2 H, m), 1.76 (3 H, s), 1.63 (3 H, s), 1.56 (3 H, s); IR (neat, *ν*_max_) cm^−1^ 2932, 1682, 1595, 1560, 1440, 1375, 1315, 1284, 1220, 1152, 1039, 1007, 924; HRMS (ESI) calcd 399.2142 for C_23_H_32_NaO_5_ [M+Na]+, found: 399.2147 *m/z.*

#### 4.1.7 5-Methyl-2-octylbenzene-1,3-diol **(10)**

3-Bis(methoxymethoxy)-5-methylbenzene (**8**) (2.00 g, 9.4 mmol) was dissolved in dry tetrahydrofuran (60 mL) and cooled to 0 °C. 2.5 M *n*-butyl lithium (4.90 mL, 12.3 mmol) was added dropwise and the reaction was left to stir for 1 h at 0 °C. Iodooctane (2.55 mL, 14.1 mmol) was added to the reaction mixture and this was stirred for 1 h before being allowed to come to room temperature. The reaction was diluted with water (300 mL) and extracted with diethyl ether (3 × 200 mL). The organic fractions were dried over sodium sulfate, filtered and concentrated under reduced pressure. Purification by flash column chromatography (25 g silica), eluting 40–60 petrol ether: ethyl acetate (100: 0) to (95: 5), gave 1,3-bis(methoxymethoxy)-5-methyl-2-octylbenzene as a colourless oil (2.54 g, 83%): R_f_ 0.79 (10% ethyl acetate/90% 40–60 petrol ether); ^1^H NMR (500 MHz, chloroform-d) *δ* 6.59 (2 H, s), 5.17 (4 H, s), 3.49 (6 H, s), 2.64 (2 H, t, *J* = 8.0 Hz), 2.30 (3 H, s), 1.50 (2 H, m), 1.32 (10 H, m), 0.89 (3 H, t, *J* = 6.8 Hz); ^13^C NMR (126 MHz, chloroform-d) *δ* 155.7, 136.6, 118.4, 108.7, 94.5, 75.4, 56.0, 31.9, 29.8, 29.7, 29.5, 29.3, 23.2, 22.7, 21.8, 14.1; IR (neat, *ν*_max_) cm^−1^ 2924, 2854, 1612, 1586, 1463, 1394, 1154, 1122, 1036, 923, 823; HRMS (ESI): calcd. 347.2198 for C_19_H_32_NaO_4_ [M+Na]^+^, found 347.2187 *m/z.*

1,3-Bis(methoxymethoxy)-5-methyl-2-octylbenzene (350 mg, 1.1 mmol) was suspended in ethylene glycol (10 mL) and heated to 160 °C by microwave irradiation for 5 h in sealed vial. The reaction mixture was diluted with water (40 mL) and extracted with diethyl ether (3 × 30 ml). The combined extracts were washed with water (30 mL), dried over magnesium sulfate, filtered and concentrated under reduced pressure. Purification by flash column chromatography (10 g silica), eluting 40–60 petrol ether: ethyl acetate (100: 0) to (70: 30), gave the title compound as an orange/white powder (220 mg, 86%, m.p. 63–66 °C): R_f_ 0.32 (10% ethyl acetate/90% 40–60 petrol ether); ^1^H NMR (500 MHz, chloroform-d) *δ* 6.23 (2 H, s), 4.69 (2 H, s), 2.59 (2 H, t, *J* = 7.9 Hz), 2.22 (3 H, s), 1.60–1.51 (2 H, m), 1.43–1.23 (10 H, m), 0.89 (3 H, t, *J* = 6.8 Hz); ^13^C NMR (126 MHz, chloroform-d) *δ* 154.4, 137.0, 112.4, 108.7, 75.4, 31.9, 29.8, 29.5, 29.3, 23.0, 22.7, 21.0, 14.1. IR (neat, *ν*_max_) cm^−1^ 3427, 3280, 2956, 2918, 2850, 1635, 1584, 1522, 1467, 1328, 1268, 1160, 1112, 829, 534; HRMS (ESI): calcd. 237.1849 for C_15_H_25_O_2_ [M+H]+, found 237.1853 *m/z*.

#### 4.1.8 3-Chloro-4,6-dihydroxy-2-methyl-5-octylbenzaldehyde **(11)**

5-Methyl-2-octyl-benzene-1,3-diol (**10**) (500 mg, 2.1 mmol) was dissolved in dry DMF (4.08 mL, 52.9 mmol) and cooled to 0 °C. Phosphorous oxychloride (0.39 mL, 4.2 mmol) was added slowly by syringe and the reaction was then allowed to come to room temperature. The reaction was followed by thin layer chromatography (20% ethyl acetate in 40–60 petroleum ether) and upon completion after 2 h the reaction was cooled to 0 °C and diluted with 1 M aqueous sodium hydroxide solution (10 mL). This was stirred for 5 min then acidified with 1 M aqueous hydrogen chloride solution (30 mL) and transferred to a separating funnel. The mixture was then extracted with diethyl ether (3 × 20 mL). The combined organic layers were washed with water (50 mL) and brine (50 mL). The organics were dried over magnesium sulfate and concentrated to dryness under reduced pressure. Purification by flash column chromatography (10 g silica), eluting 40–60 petrol ether: ethyl acetate (100: 0) to (80: 20), gave 2,4-dihydroxy-6-methyl-3-octylbenzaldehyde as an orange/white powder (559 mg, 95%, m.p. 101–103 °C): R_f_ 0.18 (10% ethyl acetate/90% 40–60 petrol ether); ^1^H NMR (500 MHz, chloroform-d) *δ* 12.63 (1 H, s), 10.09 (1 H, s), 6.20 (1 H, s), 5.52 (1 H, s), 2.60 (2 H, d, *J* = 7.1 Hz), 2.50 (3 H, s), 1.59–1.47 (2 H, m), 1.42–1.21 (10 H, m), 0.89 (3 H, d, *J* = 6.9 Hz); ^13^C NMR (126 MHz, chloroform-d) *δ* 193.0, 164.2, 161.0, 141.5, 114.0, 113.4, 110.2, 31.9, 29.7, 29.5, 29.3, 28.7, 22.6, 22.0, 17.9, 14.1; IR (neat, *ν*_max_) cm^−1^ 3111, 2916, 2849, 1605, 1509, 1466, 1284, 1250, 1216, 1135, 829, 773, 644, 567; HRMS (ESI): calcd. 265.1804 for C_16_H_25_O_3_ [M+H]^+^, found 265.1805 *m/z.*

2,4-Dihydroxy-6-methyl-3-octylbenzaldehyde (70 mg, 0.3 mmol) was dissolved in dry diethyl ether (5 mL) and sulfuryl chloride (32 μL, 0.4 mmol) added. The reaction was stirred for 1 h at room temperature before being quenched with 1 M aqueous sodium hydroxide (5 mL). The mixture was then extracted with diethyl ether (2 × 15 mL). The combined organic phases were dried over magnesium sulfate, filtered and concentrated on celite (1 g). Purification by flash column chromatography over silica (10 g) eluting 40–60 petrol ether: ethyl acetate (100: 0) to (80: 20) gave the title compound as a white powder (65 mg, 81%, m.p. 65 °C): R_f_ 0.58 (10% ethyl acetate/90% 40–60 petrol ether); ^1^H NMR (500 MHz, chloroform-d) *δ* 12.65 (1 H, s), 10.14 (1 H, s), 6.34 (1 H, s), 2.69 (2 H, d, *J* = 7.1 Hz), 2.60 (3 H, s), 1.54–1.47 (2 H, m), 1.30–1.23 (10 H, m), 0.88 (3 H, d, *J* = 6.9 Hz); ^13^C NMR (126 MHz, chloroform-d) *δ* 193.1, 162.5, 156.2, 137.2, 115.8, 113.5, 113.0, 31.9, 29.6, 29.5, 29.3, 28.4, 22.9, 22.6, 14.4, 14.1; IR (neat, *ν*_max_) cm^−1^ 3327, 2924, 2856, 1606, 1452, 1416, 1374, 1233, 1132, 791, 767, 710, 555; HRMS (ESI): calcd. 299.1414 for C_16_H_24_ClO_3_ [M+H]^+^, found 299.1421 *m/z.*

#### 4.1.9 Tert-butyl-3-(4-methyl-2,6-dioxocyclohexyl)propanoate **(12)**

5-Methylcyclohexane-1,3-dione (2.00 g, 15.9 mmol) and *t-*butyl acrylate (2.55 mL, 17.4 mmol) were dissolved in *N*,*N*-dimethylformamide (10 mL) and sodium hydride (635 mg, 15.8 mmol) added slowly. The reaction was stirred for 1 h at room temperature then heated to 80 °C for 16 h. The reaction was allowed cool to room temperature, acidified to pH 4 with acetic acid then neutralised and diluted with saturated aqueous sodium hydrogen carbonate solution (30 mL). This was extracted with ethyl acetate (3 × 30 mL). The combined organics were washed with saturated aqueous sodium hydrogen carbonate (30 mL), dried over magnesium sulfate, filtered and concentrated under reduced pressure onto celite (4 g). Purification by flash column chromatography over silica (25 g) eluting 40–60 petrol ether: ethyl acetate (100:0) to (60:40) gave the title compound as a pale yellow powder (2.94 g, 73%). Product was unstable, full characterisation after subsequent reaction to *tert*-butyl 3-(2-methoxy-4-methyl-6-oxocyclohex-1-en-1-yl)propanoate (**14**): ^1^H (500 MHz, chloroform-d) *δ* 2.49–2.36 (6 H, m), 2.18–2.02 (3 H, m), 1.42 (9 H, s), 1.02 (3 H, d, *J* = 6 Hz); HRMS (ESI) calcd 277.1410 for C_14_H_22_NaO_4_ [M+Na]^+^, found: 277.1415 *m/z*.

#### 4.1.10 5-Methyl-2-octylcyclohexane-1,3-diol **(13)**

5-Methylcyclohexane-1,3-dione (10.3 g, 81.9 mmol), octanal (8.54 mL, 54.6 mmol) and *l*-proline (1.26 g, 10.9 mmol) were dissolved in ethanol (150 mL) and stirred for 1 h. Sodium cyanoborohydride (3.43 g, 54.6 mmol) and molecular sieves (350 mg per mmol of aldehyde) were added and the reaction heated to 80 °C for 2 h. The reaction was poured onto ice (100 g) and the white solid suspension that formed was vacuum filtered. The solids were dissolved in ethyl acetate (40 mL) and washed with water (2 × 40 mL). The organics were dried over magnesium sulfate, filtered and concentrated under reduced pressure to give the title compound as a white powder (11.9 g, 91%) Product was a complex mixture of isomers and full characterisation was confirmed after successful subsequent reaction to 3-methoxy-5-methyl-2-octylcyclohex-2-enone (**15**): R_*f*_ 0.12 (20% ethyl acetate/80% 40–60 petrol ether); HRMS (ESI) calcd 277.1410 for C_15_H_27_O_2_ [M+H]^+^: found: 239.2005 *m/z*.

#### 4.1.11 Tert-butyl-3-(2-methoxy-4-methyl-6-oxocyclohex-1-en-1-yl)propanoate **(14)**

*Tert*-butyl 3-(2-hydroxy-4-methyl-6-oxo-cyclohexen-1-yl)propanoate (**12**) (16.00 g, 62.9 mmol) and dimethyl sulfate (7.14 mL, 75.5 mmol) were dissolved in tetrahydrofuran (200 mL) and sodium hydride (3.02 g, 75.5 mmol) added slowly. The reaction was stirred at room temperature for 3 h then the reaction was concentrated under reduced pressure and re-diluted with ethyl acetate (25 mL) and water (25 mL). This mixture was neutralised using excess sodium phosphate monobasic. The ethyl acetate was separated and the aqueous layer extracted with more ethyl acetate (2 × 30 mL). The combined organics were dried over magnesium sulfate, filtered and concentrated under reduced pressure onto celite (2 g). Purification by flash column chromatography over silica (100 g) eluting 40–60 petrol ether: ethyl acetate (100:0) to (80:20) gave the title compound as a clear colourless oil (15.20 g, 90%). ^1^H (600 MHz, choroform-d) *δ* 3.78 (3 H, s), 2.69–2.58 (1 H, m), 2.55–2.43 (2 H, m), 2.43–2.34 (1 H, m), 2.24–2.17 (2 H, m), 2.17–2.09 (2 H, m), 2.05–1.93 (1 H, m), 1.40 (9 H, s), 1.07 (3 H, d, J 6.1).^13^C NMR (126 MHz, chloroform-d) *δ* 197.6, 172.9, 171.5, 117.7, 79.6, 55.1, 44.7, 34.3, 33.0, 28.4, 28.1, 21.1, 17.8; IR (neat, *ν*_max_) cm^−1^ 2973, 1723, 1611, 1367, 1233, 1148, 1084, 846; HRMS (ESI): calcd. 291.1572 for C_15_H_24_NaO_4_ [M+Na]+, found 291.1572 *m/z*.

#### 4.1.12 3-Methoxy-5-methyl-2-octylcyclohex-2-enone **(15)**

5-methyl-2-octyl-cyclohexane-1,3-dione (**13**) (567 mg, 2.4 mmol) was dissolved in dry methyl alcohol (5 mL) with 3 Å molecular sieves (0.5 g) and 4-methylbenzenesulfonic acid (20 mg, 0.1 mmol) was added. The reaction was stirred for 24 h and then was concentrated onto celite (2 g). Purification by flash column chromatography over silica (10 g) eluting 40–60 petrol ether: ethyl acetate (100:0) to (90:10) gave the title compound as a yellow oil (257 mg, 43%): R_f_ 0.26 (20% ethyl acetate/80% 40–60 petrol ether); ^1^H (500 MHz, chloroform-d) *δ* 3.80 (3 H, s), 2.71–2.61 (1 H, m), 2.44 (1 H, dd, *J* = 15.7, 3.2 Hz), 2.28–2.11 (4 H, m), 2.07–1.98 (1 H, m), 1.26 (12 H, s), 1.10 (3 H, d, *J* = 6.0 Hz), 0.88 (3 H, t, *J* = 6.9 Hz); ^13^C NMR (126 MHz, chloroform-d) *δ* 198.0, 170.8, 119.6, 54.9, 44.8, 33.0, 31.8, 29.6, 29.4, 29.2, 28.6, 28.4, 22.6, 21.9, 21.1, 13.9. IR (neat, *ν*_max_) cm^−1^ 2924, 1709, 1377, 1230, 1128, 1061, 1033, 722; HRMS (ESI): calcd. 253.2168 for C_16_H_29_O_2_ [M+H]+, found 253.2163 *m/z*.

#### 4.1.13 Tert-butyl-3-(5-(hydroxymethylene)-2-methoxy-4-methyl-6-oxocyclohex-1-en-1-yl)propanoate **(16)**

Diisopropylamine (4.62 mL, 32.9 mmol) was dissolved in dry tetrahydrofuran (550 mL) and cooled to −78 °C and 2.5 M *n*-butyllithium (12.16 mL, 30.4 mmol) added. The reaction was warmed to room temperature then cooled to −78 °C and *tert*-butyl 3-(2-methoxy-4-methyl-6-oxo-cyclohexen-1-yl)propanoate (**14**) (6.8 g, 25.3 mmol) added. The reaction was stirred for 1 h at −78 °C then methyl formate (2.34 mL, 38.0 mmol) was added and it was allowed to warm to room temperature overnight. The reaction was concentrated under reduced pressure, diluted water (50 mL) and neutralised by careful addition of 1 M aqueous hydrogen chloride solution. The organics were dried over magnesium sulfate, filtered and concentrated under reduced pressure onto celite (5 g). Purification by flash column chromatography over silica (100 g) eluting 40–60 petrol ether: ethyl acetate (100:0) to (70:30) gave the title compound as a yellow oil (4.41 g, 58.6%): R_f_ 0.22 (20% ethyl acetate/80% 40–60 petrol ether); ^1^H NMR (500 MHz, chloroform-d) *δ* 7.21–7.07 (1 H, m), 3.79 (3 H, s), 2.76–2.69 (1 H, m), 2.67–2.60 (1 H, m), 2.60–2.56 (2 H, m), 2.35–2.25 (3 H, m), 1.41 (9 H, s), 1.16 (3 H, d, *J* = 6.8 Hz); ^13^C NMR (126 MHz, chloroform-d) *δ* 191.7, 172.7, 170.0, 160.3, 116.5, 111.2, 79.7, 55.2, 34.3, 32.0, 28.3, 28.1, 20.3, 17.5. IR (neat, *ν*_max_) cm^−1^ 2972, 1721, 1607, 1366, 1241, 1146, 1080, 998, 847; HRMS (ESI): calcd. 319.1521 for C_16_H_24_NaO_5_ [M+Na]^+^, found 319.1521 *m/z*.

#### 4.1.14 6-(Hydroxymethylene)-3-methoxy-5-methyl-2-octylcyclohex-2-enone **(17)**

Diisopropylamine (2.67 mL, 19.0 mmol) was dissolved in dry tetrahydrofuran (150 mL) and cooled to −78 °C and *n*-butyllithium (6.18 mL, 15.5 mmol) added. The mixture was allowed to warm to room temperature and stirred for 30 min. This was cooled to −78 °C and a solution of 3-methoxy-5-methyl-2-octyl-cyclohex-2-en-1-one (**15**) (3.00 g, 11.9 mmol) in dry tetrahydrofuran (5 mL) was added *via* syringe. This was stirred for 1 h and dry methyl formate (1.47 mL, 23.77 mmol) was added, the reaction was allowed to warm to room temperature overnight slowly in the dry ice acetone bath. The reaction mixture was concentrated under reduced pressure and diluted with ethyl acetate (30 mL) and water (30 mL). This was neutralised with 1 M aqueous hydrogen chloride, the ethyl acetate separated and washed with water (30 mL). The organics were dried over magnesium sulfate, filtered and concentrated under reduced pressure onto celite (2 g). Purification by flash column chromatography over silica (25 g) eluting 40–60 petrol ether: ethyl acetate (100:0) to (90:10) gave the title compound as a clear yellow oil (2.25 g, 68%): Rf 0.46 (20% ethyl acetate/80% 40–60 petrol ether) ^1^H (500 MHz, chloroform-d) *δ* 7.19 (1 H, d, *J* = 1.2 Hz), 3.79 (3 H, s), 2.75 (1 H, q, *J* = 6.7 Hz), 2.65 (1 H, dd, *J* = 16.6, 6.1 Hz), 2.37–2.27 (3 H, m), 1.34 (2 H, m), 1.27 (10 H, m), 1.18 (3 H, d, *J* = 6.8 Hz), 0.88 (3 H, t, *J* = 6.8 Hz); ^13^C NMR (126 MHz, chloroform-d) *δ* 191.9, 169.3, 160.5, 111.3, 55.0, 31.9, 31.8, 29.6, 29.4, 29.2, 28.6, 28.3, 22.6, 21.6, 20.4, 14.0. IR (neat, *ν*_max_) cm^−1^ 2923, 1608, 1378, 1238, 1159, 1130, 1006, 953; HRMS (ESI): calcd. 281.2117 for C_17_H_29_O_3_ [M+H]+, found 281.2113 *m/z*.

#### 4.1.15 Tert-butyl-3-(3-formyl-2-hydroxy-6-methoxy-4-methylphenyl)propanoate **(18)**

(Z)-^t^Butyl 3-(5-(hydroxymethylene)-2-methoxy-4-methyl-6-oxocyclohex-1-en-1-yl)propanoate (**16**) (1.00 g, 3.4 mmol) and 2,3-dichloro-5,6-dicyano-1,4-benzoquinone (0.92 g, 4.1 mmol) were dissolved in toluene (100 mL) and stirred for 16 h at room temperature. The reaction was diluted with diethyl ether (200 mL), the organics were washed with water (6 × 100 mL), dried over magnesium sulfate, filtered and concentrated under reduced pressure onto celite (2 g). Purification by flash column chromatography over silica (25 g) eluting 40–60 petrol ether: ethyl acetate (100:0) to (70:30) gave the title compound as a white solid (835 mg, 84%, m.p. 58–59 °C): R_f_ 0.36 (20% ethyl acetate/80% 40–60 petrol ether); ^1^H (500 MHz, chloroform-d) *δ* 12.41 (1 H, s), 10.13 (1 H, s), 6.28 (1 H, s), 3.89 (3 H, s), 2.95–2.86 (2 H, m), 2.57 (3 H, s), 2.45–2.38 (2 H, m), 1.44 (9 H, s). ^13^C NMR (126 MHz, chloroform-d) *δ* 193.1, 172.7, 164.1, 162.9, 142.3, 114.1, 113.6, 105.2, 79.9, 55.7, 34.2, 28.1, 18.4, 17.8. IR (neat, *ν*_max_) cm^−1^ 2967, 1722, 1637, 1366, 1248, 1125, 1004, 816, 644; HRMS (ESI): calcd. 317.1365 for C_16_H_22_NaO_5_ [M+Na]+, found 317.1363 *m/z*.

#### 4.1.16 2-Hydroxy-4-methoxy-6-methyl-3-octylbenzaldehyde **(19)**

6-(Hydroxymethylene)-3-methoxy-5-methyl-2-octylcyclohex-2-enone (**17**) (77 mg, 0.3 mmol) and 2,3-dichloro-5,6-dicyano-1,4-benzoquinone (73 mg, 4.1 mmol) were dissolved in toluene (20 mL) and stirred for 16 h at room temperature. The reaction was diluted with diethyl ether (50 mL), the organics were washed with water (6 × 50 mL), dried over magnesium sulfate, filtered and concentrated under reduced pressure onto celite (2 g). Purification by flash column chromatography over silica (25 g) eluting 40–60 petrol ether: ethyl acetate (100:0) to (70:30) gave the title compound as a white solid (835 mg, 84%, m.p. 58–59 °C): Rf 0.24 (20% ethyl acetate/80% 40–60 petrol ether); ^1^H (500 MHz, chloroform-d) *δ* 12.41 (1 H, s), 10.13 (1H, s), 6.28 (1H, s), 3.89 (3H, s), 2.95–2.86 (2H, m), 2.57 (3H, s), 2.45–2.38 (2H, m), 1.44 (9H, s). ^13^C NMR (126 MHz, chloroform-d) *δ* 193.1, 172.7, 164.1, 162.9, 142.3, 114.1, 113.6, 105.2, 79.9, 55.7, 34.2, 28.13, 18.4, 17.8. HRMS (ESI): calcd. 279.1955 for C_17_H_27_O_3_ [M+H]+, found 279.1948 *m/z*.

#### 4.1.17 2-Chloro-6-[(2E)-3,7-dimethylocta-2,6-dien-1-yl]-4-formyl-5-hydroxy-3-methylphenyl acetate **(20)**

To a solution of 2-chloro-5-{[(2E)-3,7-dimethylocta-2,6-dien-1-yl]oxy}-4-formyl-3-methylphenyl acetate (**5**) (757 mg, 2.07 mmol) in toluene (7.5 mL) was added Fluorosil (100–200 mesh, 2.3 g). The reaction mixture was heated at reflux for 3 h before being allowed to cool to room temperature and being filtered. The Fluorosil was washed with ethyl acetate (3 × 15 mL) and the combined filtrate was concentrated under reduced pressure. Purification by flash column chromatography (25 g silica), eluting with a gradient of petrol ether: ethyl acetate (100: 0) to (95: 5), gave the title compound as a colourless oil (212 mg, 28%): R_f_ 0.67 (5% ethyl acetate/95% 40–60 petrol ether) ^1^H NMR (500 MHz, Chloroform-*d*) *δ* 12.53 (s, 1H), 10.31 (s, 1H), 5.21–4.96 (m, 2H), 3.35–3.25 (m, 2H), 2.66 (s, 3H), 2.38 (s, 3H), 2.09–2.01 (m, 2H), 2.01–1.93 (m, 2H), 1.75 (s, 3H), 1.66 (s, 3H), 1.59 (s, 2H). LCMS (ESI) retention time 6.83 min, *m/z* 365.43/367.21.

#### 4.1.18 5-Chloro-3-[(2E)-3,7-dimethylocta-2,6-dien-1-yl]-2,4-dihydroxybenzaldehyde **(21)**

To a solution of 6-chloro-2-[(2E)-3,7-dimethylocta-2,6-dien-1-yl]-4-formyl-3-hydroxyphenyl acetate (**22**) (70.0 mg, 0.200 mmol) in tetrahydrofuran (4 mL) and methanol (1 mL) was added 1 M aqueous sodium hydroxide (1.0 mL, 1.00 mmol). The reaction mixture was stirred at room temperature for 6 h before being quenched with 1 M aqueous hydrochloric acid (5 mL) and extracted with ethyl acetate (3 × 10 mL). The combined organic fractions dried over magnesium sulfate, filtered and concentrated under reduced pressure. Purification by flash column chromatography (10 g silica), eluting with a gradient of petrol ether: ethyl acetate (100: 0) to (90: 10), gave the title compound as an off-white solid (55 mg, 89%): R_f_ 0.40 (5% ethyl acetate/95% 40–60 petrol ether) ^1^H NMR (500 MHz, Chloroform-*d*) *δ* 11.53 (s, 1H), 9.67 (s, 1H), 7.39 (s, 1H), 6.38 (s, 1H), 5.29–5.19 (m, 1H), 5.10–5.02 (m, 1H), 3.44 (d, *J* = 7.2 Hz, 2H), 2.13–2.04 (m, 2H), 2.04–1.97 (m, 2H), 1.81 (d, *J* = 1.5 Hz, 3H), 1.66 (d, *J* = 1.7 Hz, 3H), 1.58 (s, 3H).

#### 4.1.19 6-Chloro-2-[(2E)-3,7-dimethylocta-2,6-dien-1-yl]-4-formyl-3-hydroxyphenyl acetate **(22)**

To a solution of 2-chloro-5-{[(2E)-3,7-dimethylocta-2,6-dien-1-yl]oxy}-4-formylphenyl acetate (synthesised using the same method to make **5**) (430 mg, 1.22 mmol) in toluene (6.5 mL) was added Fluorosil (100–200 mesh, 2.2 g). The reaction mixture was heated at reflux for 3 h before being allowed to cool to room temperature and being filtered. The Fluorosil was washed with ethyl acetate (3 × 15 mL) and the combined filtrate was concentrated under reduced pressure. Purification 3 times by flash column chromatography (10 g silica), eluting with a gradient of petrol ether: ethyl acetate (100: 0) to (95: 5), gave the title compound as a pale yellow oil (78 mg, 18%): R_f_ 0.59 (5% ethyl acetate/95% 40–60 petrol ether) ^1^H NMR (500 MHz, Chloroform-*d*) *δ* 11.41 (s, 1H), 9.82 (s, 1H), 7.51 (s, 1H), 5.13–5.08 (m, 1H), 5.08–5.04 (m, 1H), 3.34 (d, *J* = 7.0 Hz, 2H), 2.38 (s, 3H), 2.13–2.01 (m, 2H), 2.01–1.91 (m, 2H), 1.76 (s, 3H), 1.66 (s, 3H), 1.59 (s, 3H). LCMS (ESI) retention time 6.64 min, *m/z* 351.59/353.19.

#### 4.1.20 3-[(2E)-3,7-Dimethylocta-2,6-dien-1-yl]-2,4-dihydroxy-6-methylbenzaldehyde **(23)**

To a solution of 2-[(2E)-3,7-dimethylocta-2,6-dien-1-yl]-4-formyl-3-hydroxy-5-methylphenyl acetate (**24**) (6.0 mg, 0.018 mmol) in tetrahydrofuran (0.5 mL) and methanol (0.12 mL) was added 4 M aqueous sodium hydroxide (9 μL, 1.00 mmol). The reaction mixture was stirred at room temperature for 3 h before being acidified to pH 2 with 1 M aqueous hydrochloric acid and extracted with ethyl acetate (3 × 3 mL). The combined organic fractions dried over magnesium sulfate, filtered and concentrated under reduced pressure. Purification by flash column chromatography (1 g silica), eluting with a gradient of petrol ether: ethyl acetate (100: 0) to (90: 10), gave the title compound as a white solid (2.7 mg, 51%): R_f_ 0.15 (10% ethyl acetate/90% 40–60 petrol ether) ^1^H NMR (500 MHz, Chloroform-*d*) *δ* 12.78 (s, 1H), 10.09 (s, 1H), 6.22 (s, 1H), 6.15 (s, 1H), 5.31–5.22 (m, 1H), 5.12–5.00 (m, 1H), 3.42 (d, *J* = 7.2 Hz, 2H), 2.51 (s, 3H), 2.19–2.01 (m, 4H), 1.82 (d, *J* = 1.5 Hz, 3H), 1.69 (d, *J* = 1.6 Hz, 3H), 1.60 (s, 3H). LCMS (ESI) retention time 5.73 min, *m/z* 289.67.

#### 4.1.21 2-[(2E)-3,7-Dimethylocta-2,6-dien-1-yl]-4-formyl-3-hydroxy-5-methylphenyl acetate **(24)**

To a solution of 3-{[(2E)-3,7-dimethylocta-2,6-dien-1-yl]oxy}-4-formyl-5-methylphenyl acetate (synthesised using the same method to make **5**) (300 mg, 0.908 mmol) in toluene (9.0 mL) was added Fluorosil (100–200 mesh, 3.0 g). The reaction mixture was heated at reflux for 3 h before being allowed to cool to room temperature and being filtered. The Fluorosil was washed with ethyl acetate (3 × 10 mL) and the combined filtrate was concentrated under reduced pressure. Purification by flash column chromatography (10 g silica), eluting with a gradient of petrol ether: ethyl acetate (100: 0) to (80: 20) then by prep HPLC, gave the title compound as a pale yellow oil (12 mg, 4%): R_f_ 0.28 (10% ethyl acetate/90% 40–60 petrol ether) ^1^H NMR (500 MHz, Chloroform-*d*) *δ* 12.45 (s, 1H), 10.25 (s, 1H), 6.47 (s, 1H), 5.17–5.09 (m, 1H), 5.08–5.01 (m, 1H), 3.28 (d, *J* = 7.0 Hz, 2H), 2.57 (s, 3H), 2.31 (s, 3H), 2.10–2.00 (m, 2H), 2.01–1.95 (m, 2H), 1.75 (s, 3H), 1.65 (d, *J* = 1.6 Hz, 3H), 1.58 (s, 3H). LCMS (ESI) retention time 6.44 min, *m/z* 331.72.

#### 4.1.22 3-[(2E)-3,7-Dimethylocta-2,6-dien-1-yl]-2,4-dihydroxybenzaldehyde **(25)**

To a solution of 2-[(2E)-3,7-dimethylocta-2,6-dien-1-yl]-4-formyl-3-hydroxyphenyl acetate (**26**) (130 mg, 0.411 mmol) in tetrahydrofuran (4.0 mL) and methanol (1.0 mL) was added 4 M aqueous sodium hydroxide (308 μL, 1.23 mmol). The reaction mixture was stirred at room temperature for 3 h before being diluted with deionised water (10 mL), acidified to pH 1 with 1 M aqueous hydrochloric acid and extracted with ethyl acetate (3 × 10 mL). The combined organic fractions dried over magnesium sulfate, filtered and concentrated under reduced pressure. Purification by flash column chromatography (10 g silica), eluting with a gradient of petrol ether: ethyl acetate (100: 0) to (80: 20), gave the title compound as a cream coloured solid (83 mg, 73%): R_f_ 0.36 (20% ethyl acetate/80% 40–60 petrol ether) ^1^H NMR (500 MHz, Chloroform-*d*) *δ* 11.79 (s, 1H), 9.70 (s, 1H), 7.32 (d, *J* = 8.7 Hz, 1H), 6.49 (d, *J* = 8.5 Hz, 1H), 6.21 (s, 1H), 5.34–5.21 (m, 1H), 5.11–4.98 (m, 1H), 3.47 (d, *J* = 7.2 Hz, 2H), 2.18–2.02 (m, 4H), 1.83 (s, 3H), 1.68 (d, *J* = 1.7 Hz, 3H), 1.60 (s, 3H). LCMS (ESI) retention time 5.57 min, *m/z* 275.64.

#### 4.1.23 2-[(2E)-3,7-Dimethylocta-2,6-dien-1-yl]-4-formyl-3-hydroxyphenyl acetate **(26)**

To a solution of 3-{[(2E)-3,7-dimethylocta-2,6-dien-1-yl]oxy}-4-formylphenyl acetate (synthesised using the same method to make **5**) (520 mg, 1.64 mmol) in toluene (7.5 mL) was added Fluorosil (100–200 mesh, 2.6 g). The reaction mixture was heated at reflux for 3 h before being allowed to cool to room temperature and being filtered. The Fluorosil was washed with ethyl acetate (3 × 20 mL) and the combined filtrate was concentrated under reduced pressure. Purification 2 times by flash column chromatography (10 g silica), eluting with a gradient of petrol ether: ethyl acetate (100: 0) to (90: 10), gave the title compound as a pale yellow oil (133 mg, 26%): R_f_ 0.50 (10% ethyl acetate/90% 40–60 petrol ether) ^1^H NMR (500 MHz, Chloroform-*d*) *δ* 11.54 (s, 1H), 9.85 (s, 1H), 7.44 (d, *J* = 8.5 Hz, 1H), 6.76 (d, *J* = 8.4 Hz, 1H), 5.17–5.10 (m, 1H), 5.09–5.02 (m, 1H), 3.33 (d, *J* = 7.0 Hz, 2H), 2.33 (s, 3H), 2.10–2.02 (m, 2H), 2.02–1.94 (m, 2H), 1.77 (s, 3H), 1.65 (d, *J* = 1.8 Hz, 3H), 1.58 (s, 3H).

#### 4.1.24 5-Chloro-3-[(2E)-3,7-dimethylocta-2,6-dien-1-yl]-2-hydroxybenzaldehyde **(27)**

To a solution of 5-chloro-2-{[(2E)-3,7-dimethylocta-2,6-dien-1-yl]oxy}benzaldehyde (synthesised using the same method to make **5**) (300 mg, 1.02 mmol) in toluene (5.0 mL) was added Fluorosil (100–200 mesh, 3.0 g). The reaction mixture was heated at reflux for 3 h before being allowed to cool to room temperature and being filtered. The Fluorosil was washed with ethyl acetate (3 × 10 mL) and the combined filtrate was concentrated under reduced pressure. Purification 2 times by flash column chromatography (10 g silica), eluting with a gradient of petrol ether: ethyl acetate (100: 0) to (99: 1), gave the title compound as a pale yellow oil (45 mg, 15%): R_f_ 0.12 (100% 40–60 petrol ether) ^1^H NMR (500 MHz, Chloroform-*d*) *δ* 11.22 (s, 1H), 9.84 (d, *J* = 0.8 Hz, 1H), 7.39 (d, *J* = 2.6 Hz, 1H), 7.35 (d, *J* = 2.6 Hz, 1H), 5.37–5.23 (m, 1H), 5.17–5.05 (m, 1H), 3.37 (d, *J* = 7.4 Hz, 2H), 2.21–2.02 (m, 4H), 1.76–1.65 (m, 6H), 1.62 (s, 3H).

#### 4.1.25 3-[(2E)-3,7-Dimethylocta-2,6-dien-1-yl]-2,4,6-trihydroxybenzaldehyde **(28)**

To a solution of 2,4,6-trihydroxybenzaldehyde (500 mg, 3.24 mmol) and geranyl bromide (536 μL, 2.70 mmol) in acetone (30 mL) was added potassium carbonate (224 mg, 1.62 mmol). The reaction mixture was heated at reflux for 5 h before being allowed to cool to room temperature and being concentrated under reduced pressure. Purification by flash column chromatography (10 g silica), eluting with a gradient of petrol ether: ethyl acetate (100: 0) to (60: 40), gave the title compound as a yellow solid (102 mg, 13%): R_f_ 0.36 (40% ethyl acetate/60% 40–60 petrol ether) ^1^H NMR (500 MHz, Chloroform-*d*) *δ* 12.25 (s, 1H), 10.04 (s, 1H), 7.65 (d, 1H), 7.22 (s, 1H), 5.90 (s, 1H), 5.29–5.23 (m, 1H), 5.05 (t, *J* = 6.7 Hz, 1H), 3.33 (d, *J* = 7.3 Hz, 2H), 2.21–1.97 (m, 4H), 1.80 (s, 3H), 1.67 (s, 3H), 1.59 (s, 3H).

#### 4.1.26 3-Chloro-5-[(2E)-3,7-dimethylocta-2,6-dien-1-yl]-4,6-dihydroxy-2-methylbenzonitrile **(29)**

To a solution of 5-chloro-7-[(2E)-3,7-dimethylocta-2,6-dien-1-yl]-4-methyl-1,2-benzoxazol-6-ol (synthesised using the same method to make **31**) (45 mg, 0.141 mmol) in ethanol (4.5 mL) at room temperature was added 1 M aqueous sodium hydroxide (1.4 mL, 1.4 mmol). The reaction mixture was stirred at room temperature for 1 h before being acidified to pH 1 with 1 M aqueous hydrochloric acid. Ethyl acetate (10 mL) was added and the phases were separated. The aqueous phase was extracted with ethyl acetate (2 × 10 mL) and the combined organic fractions were dried over magnesium sulfate, filtered and concentrated under reduced pressure. Purification by flash column chromatography (5 g silica), eluting with a gradient of petrol ether: ethyl acetate (100: 0) to (90: 10), gave the title compound as a colourless oil (17 mg, 38%): R_f_ 0.40 (10% ethyl acetate/90% 40–60 petrol ether) ^1^H NMR (500 MHz, Chloroform-*d*) *δ* 6.24 (s, 1H), 6.17 (s, 1H), 5.28–5.19 (m, 1H), 5.09–5.02 (m, 1H), 3.46 (d, *J* = 7.2 Hz, 2H), 2.52 (s, 3H), 2.21–2.02 (m, 4H), 1.86–1.78 (m, 3H), 1.70 (d, *J* = 1.3 Hz, 3H), 1.61 (d, *J* = 1.6 Hz, 3H). LCMS (ESI) retention time 5.43 min, *m/z* 320.15/322.07.

#### 4.1.27 2-Chloro-6-[(2E)-3,7-dimethylocta-2,6-dien-1-yl]-5-hydroxy-3,4-dimethylphenyl acetate **(30)**

To a solution of 2-chloro-6-[(2E)-3,7-dimethylocta-2,6-dien-1-yl]-4-formyl-5-hydroxy-3-methylphenyl acetate (**20**) (100 mg, 0.274 mmol) in dichloromethane (5 mL) was added triethylamine (76 μL, 0.548 mmol) and acetyl chloride (39 μL, 0.548 mmol) at room temperature. The reaction mixture was stirred at room temperature for 3 h before being concentrated under reduced pressure. Purification by flash column chromatography (10 g silica), eluting with a gradient of petrol ether: ethyl acetate (100: 0) to (80: 20), 3-(acetyloxy)-6-chloro-2-[(2E)-3,7-dimethylocta-2,6-dien-1-yl]-4-formyl-5-methylphenyl acetate as a pale yellow oil (30 mg, 27%): R_f_ 0.40 (10% ethyl acetate/90% 40–60 petrol ether) ^1^H NMR (500 MHz, Chloroform-*d*) *δ* 10.27 (s, 1H), 5.12–5.01 (m, 1H), 5.01–4.91 (m, 1H), 3.21 (d, *J* = 6.6 Hz, 2H), 2.68 (s, 3H), 2.37 (s, 3H), 2.36 (s, 3H), 2.09–2.01 (m, 2H), 2.01–1.94 (m, 2H), 1.73 (d, *J* = 1.5 Hz, 3H), 1.67 (d, *J* = 1.7 Hz, 3H), 1.59 (d, *J* = 1.4 Hz, 3H). LCMS (ESI) retention time 6.44 min, *m/z* no ionisation.

To a solution of 3-(acetyloxy)-6-chloro-2-[(2E)-3,7-dimethylocta-2,6-dien-1-yl]-4-formyl-5-methylphenyl acetate (30 mg, 0.073 mmol) in tetrahydrofuran (1.0 mL) was added sodium borohydride (11 mg, 0.295 mmol). The reaction mixture was stirred at room temperature for 3 h before being diluted with deionised water (1 mL), quenched with 1 M aqueous hydrochloric acid (3 mL) and extracted with ethyl acetate (3 × 10 mL). The combined organic extracts were dried over magnesium sulfate, filtered and concentrated under reduced pressure. Purification by flash column chromatography (10 g silica), eluting with a gradient of petrol ether: ethyl acetate (100: 0) to (90: 10), gave the title compound as an off-white solid (15 mg, 58%): R_f_ 0.57 (10% ethyl acetate/90% 40–60 petrol ether) ^1^H NMR (500 MHz, Chloroform-*d*) *δ* 5.43 (s, 1H), 5.22–5.12 (m, 1H), 5.09–4.97 (m, 1H), 3.28 (d, *J* = 7.6 Hz, 2H), 2.36 (s, 3H), 2.33 (s, 3H), 2.18 (s, 3H), 2.15–2.04 (m, 4H), 1.81 (s, 3H), 1.69 (d, *J* = 1.4 Hz, 3H), 1.61 (s, 3H). LCMS (ESI) retention time 6.43 min, *m/z* no ionisation.

#### 4.1.28 5-Chloro-3-[(2E)-3,7-dimethylocta-2,6-dien-1-yl]-2,4-dihydroxybenzonitrile **(31)**

To a solution of triphenylphosphine (42 mg, 0.162 mmol) in dichloromethane (2 mL) was added 2,3-dichloro-5,6-dicyano-*p*-benzoquinone (37 mg, 0.162 mmol). The dark reaction mixture was stirred at room temperature for 1 min during which time the colour faded. 4-Chloro-2-2E)-3,7-dimethylocta-2,6-dien-1-yl]-6-[(hydroxyimino)methyl]benzene-1,3-diol (**32**) (35 mg, 0.108 mmol) in dichloromethane (0.5 mL) was added to the reaction mixture in 1 portion and this was stirred for 1 min before being concentrated under reduced pressure. Purification by flash column chromatography (10 g silica), eluting with a gradient of petrol ether: ethyl acetate (100: 0) to (90: 10), gave 5-chloro-7-[(2E)-3,7-dimethylocta-2,6-dien-1-yl]-1,2-benzoxazol-6-ol as a white solid (24 mg, 72%).

To a solution of 5-chloro-7-[(2E)-3,7-dimethylocta-2,6-dien-1-yl]-1,2-benzoxazol-6-ol in ethanol (2.1 mL) at room temperature was added 1 M aqueous sodium hydroxide (0.69 mL, 0.69 mmol). The reaction mixture was stirred at room temperature for 1 h before being acidified to pH 1 with 1 M aqueous hydrochloric acid. Ethyl acetate (10 mL) was added and the phases were separated. The aqueous phase was extracted with ethyl acetate (2 × 10 mL) and the combined organic fractions were dried over magnesium sulfate, filtered and concentrated under reduced pressure. Purification by flash column chromatography (5 g silica), eluting with a gradient of petrol ether: ethyl acetate (100: 0) to (90: 10), gave the title compound as a light green oil (12 mg, 57%): R_f_ 0.09 (5% ethyl acetate/95% 40–60 petrol ether) ^1^H NMR (500 MHz, Chloroform-*d*) *δ* 7.38 (s, 1H), 6.31 (s, 1H), 6.11 (s, 1H), 5.33–5.17 (m, 1H), 5.11–4.97 (m, 1H), 3.49 (d, *J* = 7.4 Hz, 3H), 2.20–2.01 (m, 4H), 1.82 (s, 3H), 1.70 (s, 3H), 1.61 (s, 3H). LCMS (ESI) retention time 4.98 min, *m/z* 306.09.

#### 4.1.29 4-Chloro-2-[(2E)-3,7-dimethylocta-2,6-dien-1-yl]-6-[(hydroxyimino)methyl]benzene-1,3-diol **(32)**

To a solution of 3-[(2E)-3,7-dimethylocta-2,6-dien-1-yl]-2,4-dihydroxy-6-methylbenzaldehyde (**23**) (44 mg, 0.142 mmol) in ethanol/deionised water/tetrahydrofuran (3:2:2, 0.5 mL) was added sodium acetate (17 mg, 0.214 mmol) and hydroxylamine hydrochloride (11 mg, 0.157 mmol). The reaction mixture was stirred for 16 h before being concentrated under reduced pressure. Purification by flash column chromatography (5 g silica), eluting with a gradient of petrol ether: ethyl acetate (100: 0) to (90: 10), gave the title compound as a white solid (37 mg, 80%): R_f_ 0.30 (5% ethyl acetate/95% 40–60 petrol ether) ^1^H NMR (500 MHz, Chloroform-*d*) *δ* 9.31 (s, 1H), 8.10 (s, 1H), 7.08 (s, 1H), 7.02 (s, 1H), 5.88 (s, 1H), 5.35–5.18 (m, 1H), 5.14–5.00 (m, 1H), 3.46 (d, *J* = 7.2 Hz, 2H), 2.12–2.04 (m, 2H), 2.04–1.96 (m, 2H), 1.81 (d, *J* = 1.5 Hz, 3H), 1.66 (s, 3H), 1.59 (s, 3H). LCMS (ESI) retention time 5.40 min, *m/z* 324.09/326.11.

#### 4.1.30 3-Chloro-5-[(2E)-3,7-dimethylocta-2,6-dien-1-yl]-4,6-dimethoxy-2-methylbenzaldehyde **(33)**

To a solution of 3-chloro-5-[(2E)-3,7-dimethylocta-2,6-dien-1-yl]-4,6-dihydroxy-2-methylbenzaldehyde (**6**) (30 mg, 0.0929 mmol) and potassium carbonate (128 mg, 0.929 mmol) in acetone (2 ml) was added methyl iodide (57 μL, 0.929 mmol). The reaction mixture was stirred at room temperature for 16 h before being concentrated under reduced pressure. The residue was dissolved in ethyl acetate (5 mL) and deionised water (5 mL). The phases were separated and the aqueous phase was extracted with ethyl acetate (2 × 5 mL). The combined organic extracts were dried over magnesium sulfate, filtered and concentrated under reduced pressure. Purification by flash column chromatography (5 g silica), eluting with a gradient of petrol ether: ethyl acetate (100: 0) to (95: 5), gave the title compound as a yellow oil (16 mg, 49%): R_f_ 0.71 (5% ethyl acetate/95% 40–60 petrol ether) ^1^H NMR (500 MHz, Chloroform-*d*) *δ* 10.43 (s, 1H), 5.24–5.12 (m, 1H), 5.12–5.00 (m, 1H), 3.88 (s, 3H), 3.83 (s, 3H), 3.42 (d, *J* = 7.0 Hz, 2H), 2.65 (s, 3H), 2.12–2.04 (m, 2H), 2.04–1.96 (m, 2H), 1.80 (s, 3H), 1.64 (s, 3H), 1.60–1.55 (m, 3H).

#### 4.1.31 3-Chloro-4-{[(2E)-3,7-dimethylocta-2,6-dien-1-yl]oxy}-6-hydroxy-2-methylbenzaldehyde **(34)**

To a solution of 4-chloro-5-{[(2E)-3,7-dimethylocta-2,6-dien-1-yl]oxy}-2-formyl-3-methylphenyl acetate (50 mg, 0.137 mmol) in tetrahydrofuran (2 mL) and methanol (0.5 ml) was added 4 M aqueous sodium hydroxide (68 μL, 0.274 mmol). The reaction mixture was stirred at room temperature for 2 h before being diluted with deionised water (2 mL) and acidified to pH 2 with 1 M aqueous hydrochloric acid. The reaction mixture was extracted with ethyl acetate (3 × 3 mL). The combined organic extracts were dried over magnesium sulfate, filtered and concentrated under reduced pressure. Purification by flash column chromatography (10 g silica), eluting with a gradient of petrol ether: ethyl acetate (100: 0) to (95: 5), gave the title compound as a white solid (31 mg, 70%): R_f_ 0.16 (5% ethyl acetate/95% 40–60 petrol ether) ^1^H NMR (500 MHz, Chloroform-*d*) *δ* 12.59 (s, 1H), 10.16 (s, 1H), 6.36 (s, 1H), 5.53–5.43 (m, 1H), 5.14–5.03 (m, 1H), 4.67 (d, *J* = 6.5 Hz, 2H), 2.63 (s, 3H), 2.22–2.05 (m, 4H), 1.76 (d, *J* = 1.4 Hz, 3H), 1.68 (d, *J* = 1.6 Hz, 3H), 1.61 (d, *J* = 1.5 Hz, 3H). LCMS (ESI) retention time 6.88 min, *m/z* 323.72/325.95.

#### 4.1.32 3-Chloro-6-{[(2E)-3,7-dimethylocta-2,6-dien-1-yl]oxy}-4-hydroxy-2-methylbenzaldehyde **(35)**

To a solution of 2-chloro-5-{[(2E)-3,7-dimethylocta-2,6-dien-1-yl]oxy}-4-formyl-3-methylphenyl acetate (**5**) (50 mg, 0.137 mmol) in tetrahydrofuran (2 mL) and methanol (0.5 ml) was added 4 M aqueous sodium hydroxide (68 μL, 0.274 mmol). The reaction mixture was stirred at room temperature for 2 h before being diluted with deionised water (2 mL) and acidified to pH 2 with 1 M aqueous hydrochloric acid. The reaction mixture was extracted with ethyl acetate (3 × 3 mL). The combined organic extracts were dried over magnesium sulfate, filtered and concentrated under reduced pressure. Purification by flash column chromatography (10 g silica), eluting with a gradient of petrol ether: ethyl acetate (100: 0) to (90: 10), gave the title compound as a white solid (39 mg, 88%): R_f_ 0.23 (10% ethyl acetate/90% 40–60 petrol ether) ^1^H NMR (500 MHz, Chloroform-*d*) *δ* 10.50 (s, 1H), 6.56 (s, 1H), 6.14 (s, 1H), 5.51–5.43 (m, 1H), 5.14–5.04 (m, 1H), 4.61 (d, *J* = 6.5 Hz, 2H), 2.69 (s, 3H), 2.21–2.04 (m, 3H), 1.75 (d, *J* = 1.5 Hz, 3H), 1.69 (d, *J* = 1.8 Hz, 3H), 1.62 (s, 3H). LCMS (ESI) retention time 5.88 min, *m/z* 323.22/325.21.

## Appendix A Supplementary data

LEP-0117-01 LEP-0117-01

LEP-0123-01 LEP-0123-01

LEP-0124-01 LEP-0124-01

LEP-0131-01 LEP-0131-01

LEP-0173-01 LEP-0173-01

LEP-0183-01 LEP-0183-01

LEP-0221-01 LEP-0221-01
