# Supplementary figures and images for: African trypanosomiasis: Synthesis & SAR enabling novel drug discovery of ubiquinol mimics for trypanosome alternative oxidase
Source: Eur J Med Chem. 2017 Dec 1;141:676–89. doi: 10.1016/j.ejmech.2017.09.067 (PMC5697954; doi:10.1016/j.ejmech.2017.09.067)

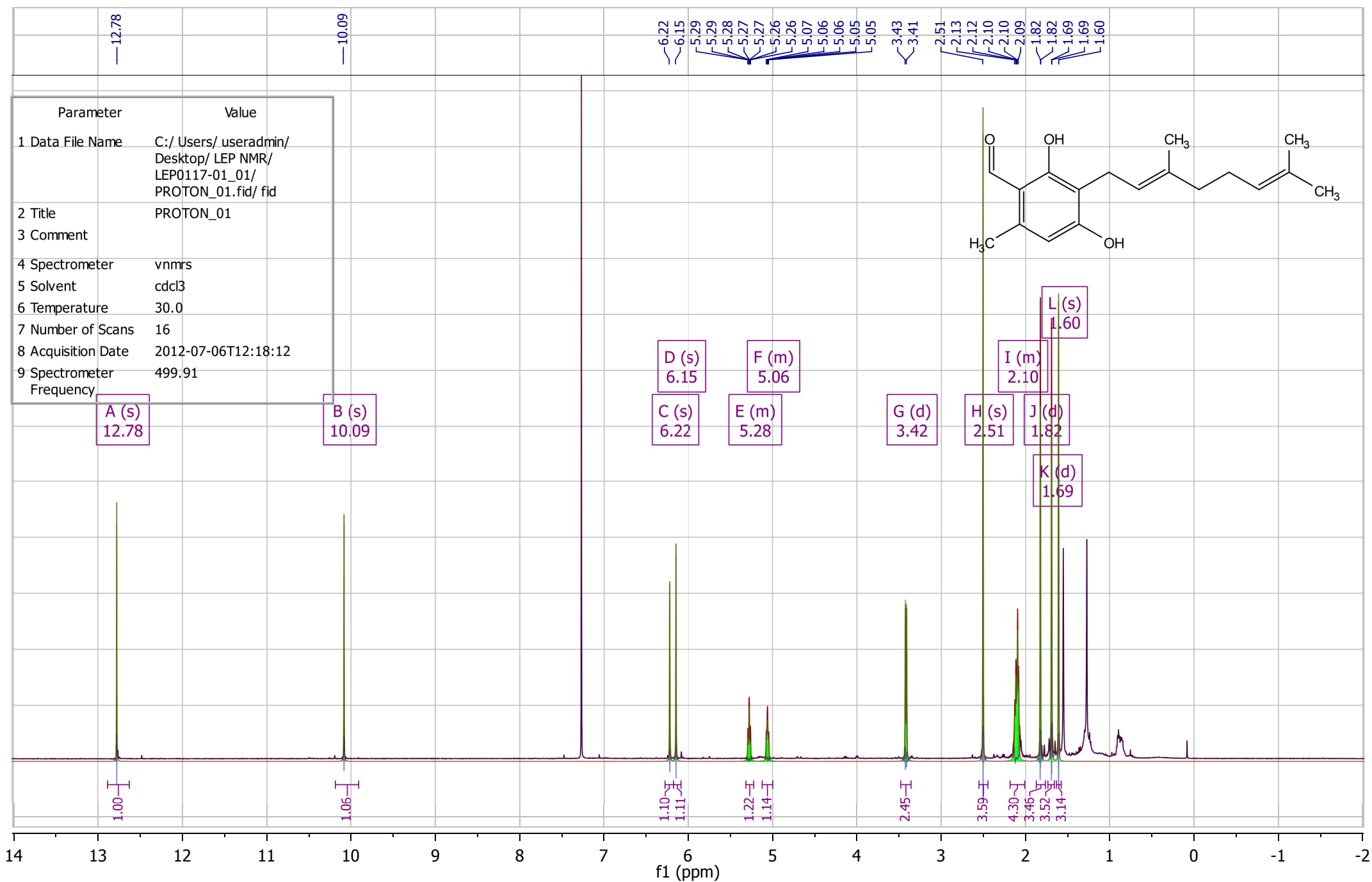

<sup>1</sup>H NMR (500 MHz, Chloroform-*d*)  $\delta$  12.78 (s, 1H), 10.09 (s, 1H), 6.22 (s, 1H), 6.15 (s, 1H), 5.31 – 5.22 (m, 1H), 5.12 – 5.00 (m, 1H), 3.42 (d,  $J$  = 7.2 Hz, 2H), 2.51 (s, 3H), 2.19 – 2.01 (m, 4H), 1.82 (d,  $J$  = 1.5 Hz, 3H), 1.69 (d,  $J$  = 1.6 Hz, 3H), 1.60 (s, 3H).

Supplement: LEP-0117-01 [file mmc1.pdf]

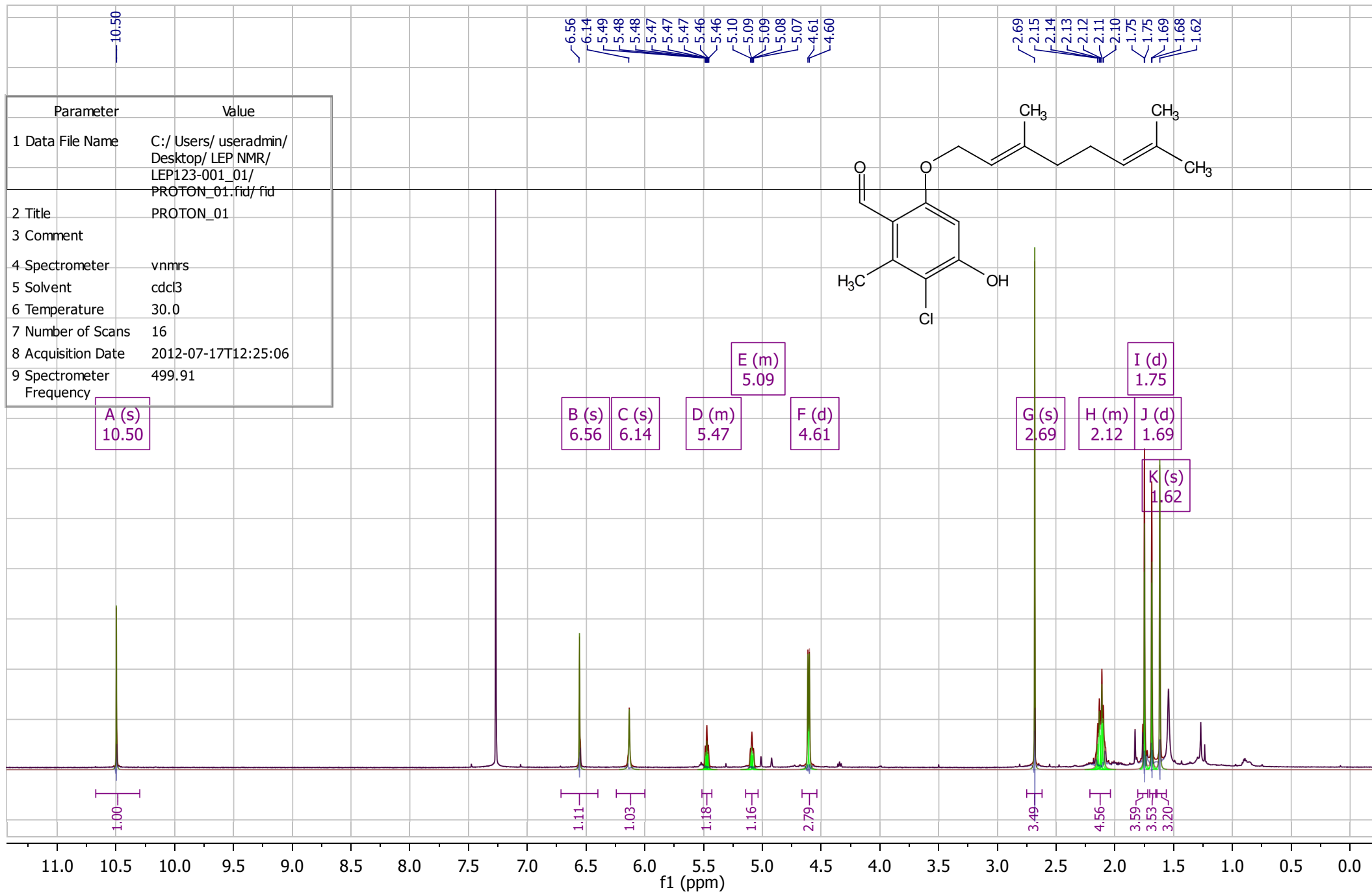

$^1\text{H}$  NMR (500 MHz, Chloroform- $d$ )  $\delta$  10.50 (s, 1H), 6.56 (s, 1H), 6.14 (s, 1H), 5.51 – 5.43 (m, 1H), 5.14 – 5.04 (m, 1H), 4.61 (d,  $J$  = 6.5 Hz, 2H), 2.69 (s, 3H), 2.21 – 2.04 (m, 3H), 1.75 (d,  $J$  = 1.5 Hz, 3H), 1.69 (d,  $J$  = 1.8 Hz, 3H), 1.62 (s, 3H).

Supplement: LEP-0123-01 [file mmc2.pdf]

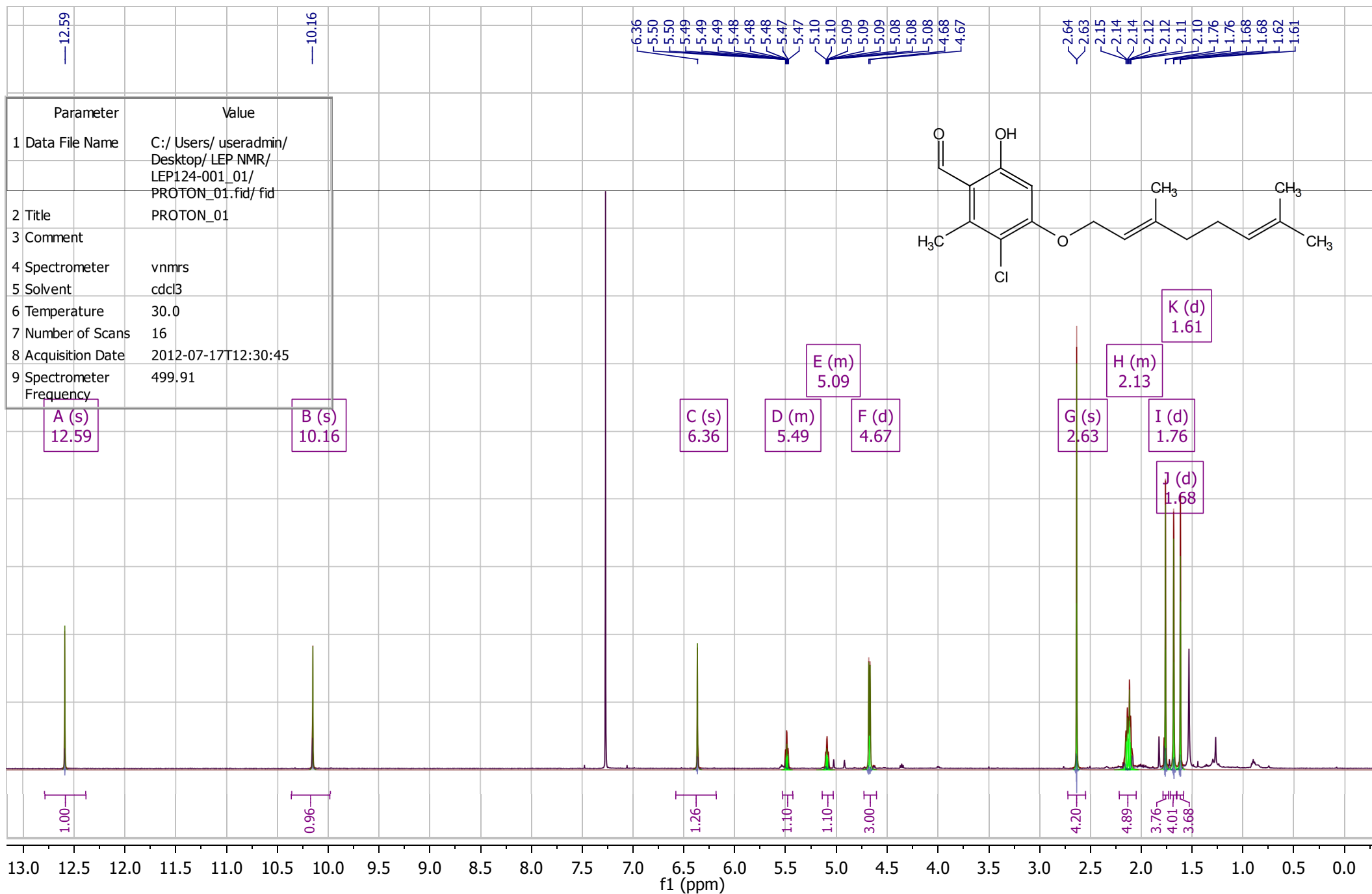

<sup>1</sup>H NMR (500 MHz, Chloroform-*d*) δ 12.59 (s, 1H), 10.16 (s, 1H), 6.36 (s, 1H), 5.53 – 5.43 (m, 1H), 5.14 – 5.03 (m, 1H), 4.67 (d, *J* = 6.5 Hz, 2H), 2.63 (s, 3H), 2.22 – 2.05 (m, 4H), 1.76 (d, *J* = 1.4 Hz, 3H), 1.68 (d, *J* = 1.6 Hz, 3H), 1.61 (d, *J* = 1.5 Hz, 3H).

Supplement: LEP-0124-01 [file mmc3.pdf]

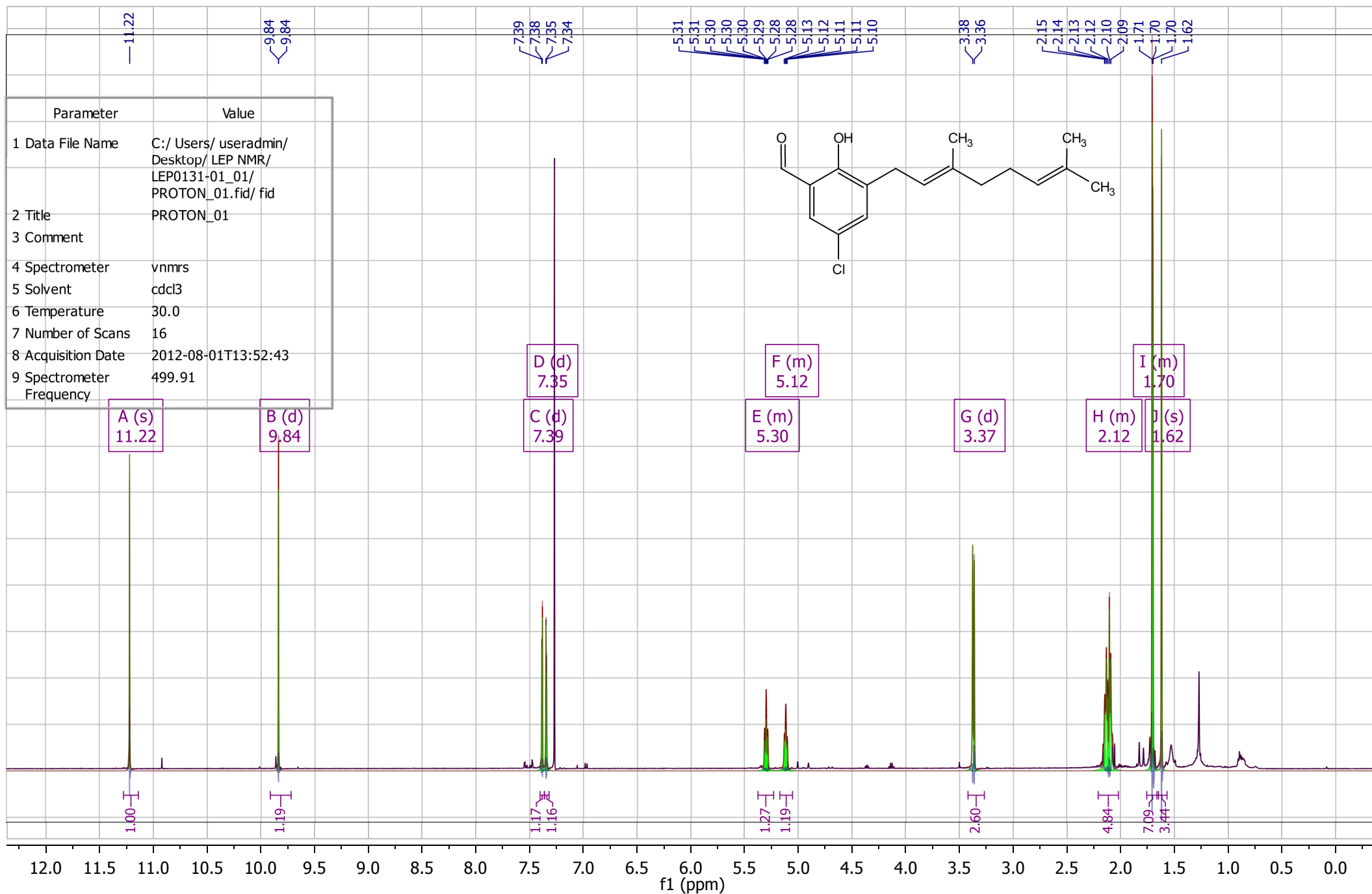

$^1\text{H}$  NMR (500 MHz, Chloroform- $d$ )  $\delta$  11.22 (s, 1H), 9.84 (d,  $J$  = 0.8 Hz, 1H), 7.39 (d,  $J$  = 2.6 Hz, 1H), 7.35 (d,  $J$  = 2.6 Hz, 1H), 5.37 – 5.23 (m, 1H), 5.17 – 5.05 (m, 1H), 3.37 (d,  $J$  = 7.4 Hz, 2H), 2.21 – 2.02 (m, 4H), 1.76 – 1.65 (m, 6H), 1.62 (s, 3H).

Supplement: LEP-0131-01 [file mmc4.pdf]

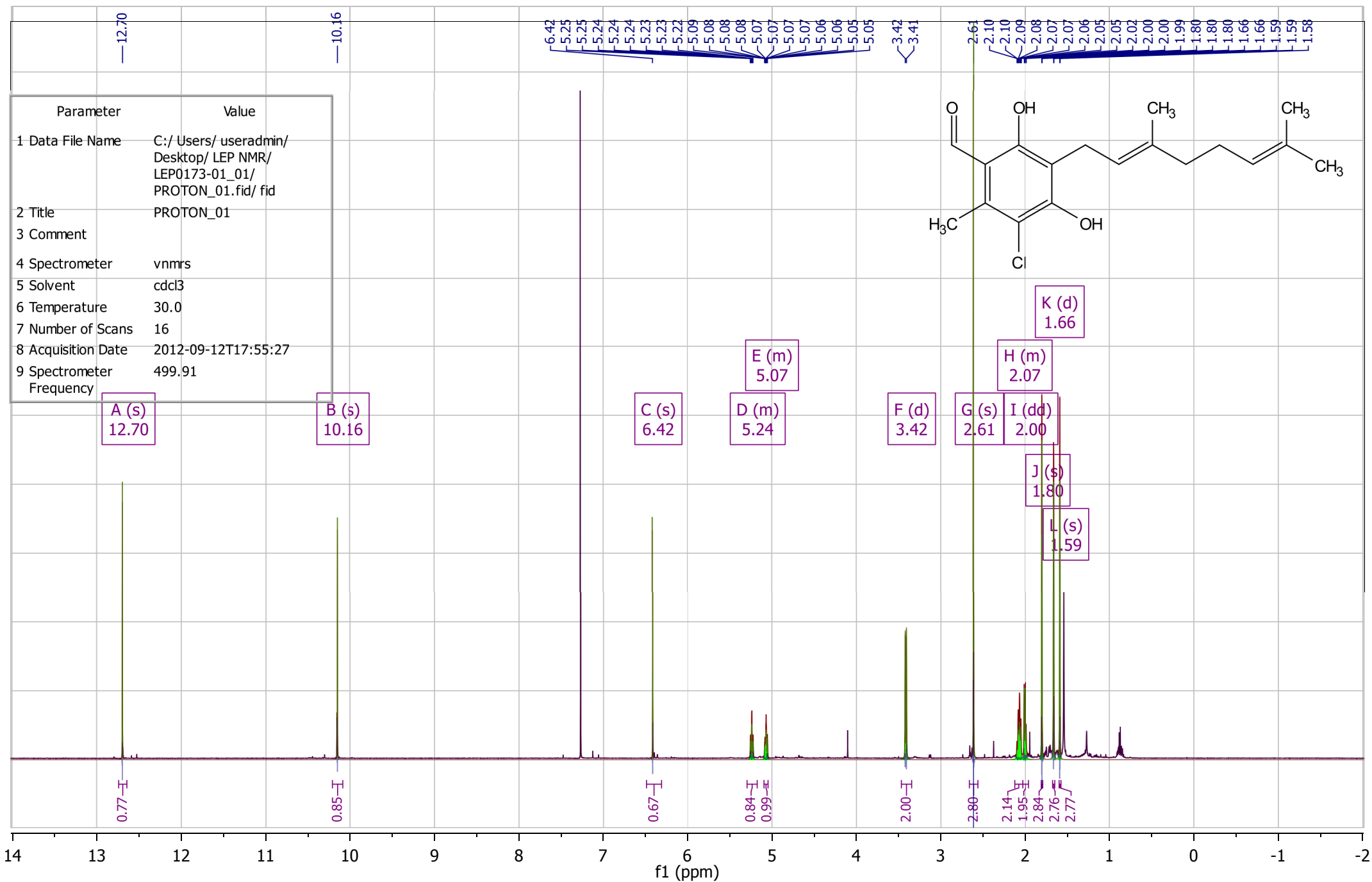

$^1\text{H}$  NMR (500 MHz, Chloroform-*d*)  $\delta$  12.70 (s, 1H), 10.16 (s, 1H), 6.42 (s, 1H), 5.29 – 5.17 (m, 1H), 5.09 – 5.05 (m, 1H), 3.42 (d,  $J$  = 7.1 Hz, 2H), 2.61 (s, 3H), 2.13 – 2.03 (m, 2H), 2.00 (dd,  $J$  = 9.0, 6.1 Hz, 2H), 1.80 (s, 3H), 1.66 (d,  $J$  = 1.4 Hz, 3H), 1.59 (s, 3H).

Supplement: LEP-0173-01 [file mmc5.pdf]

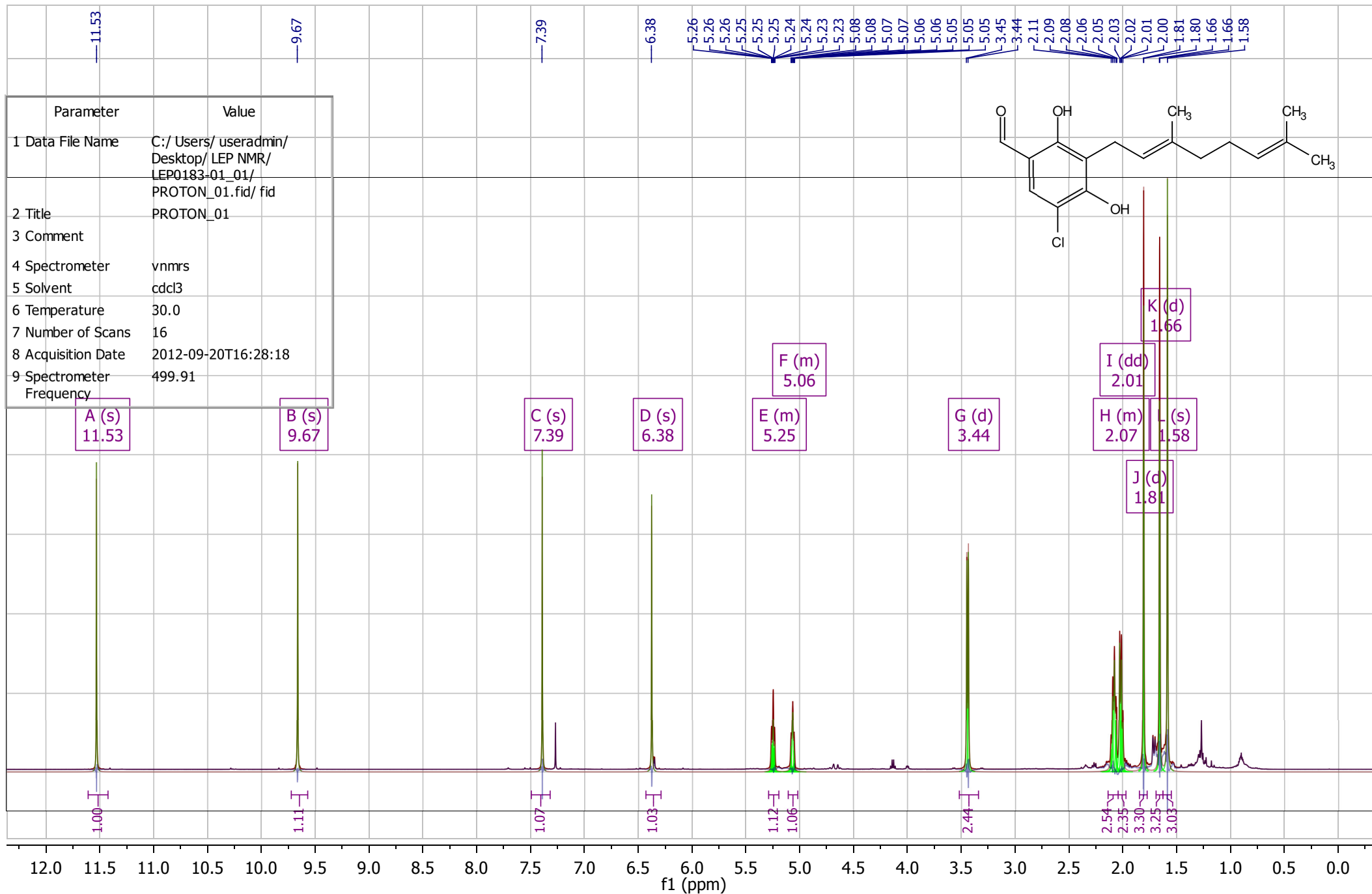

$^1\text{H}$  NMR (500 MHz, Chloroform- $d$ )  $\delta$  11.53 (s, 1H), 9.67 (s, 1H), 7.39 (s, 1H), 6.38 (s, 1H), 5.29 – 5.19 (m, 1H), 5.10 – 5.02 (m, 1H), 3.44 (d,  $J$  = 7.2 Hz, 2H), 2.13 – 2.04 (m, 2H), 2.01 (dd,  $J$  = 9.0, 6.2 Hz, 2H), 1.81 (d,  $J$  = 1.5 Hz, 3H), 1.66 (d,  $J$  = 1.7 Hz, 3H), 1.58 (s, 3H).

Supplement: LEP-0183-01 [file mmc6.pdf]

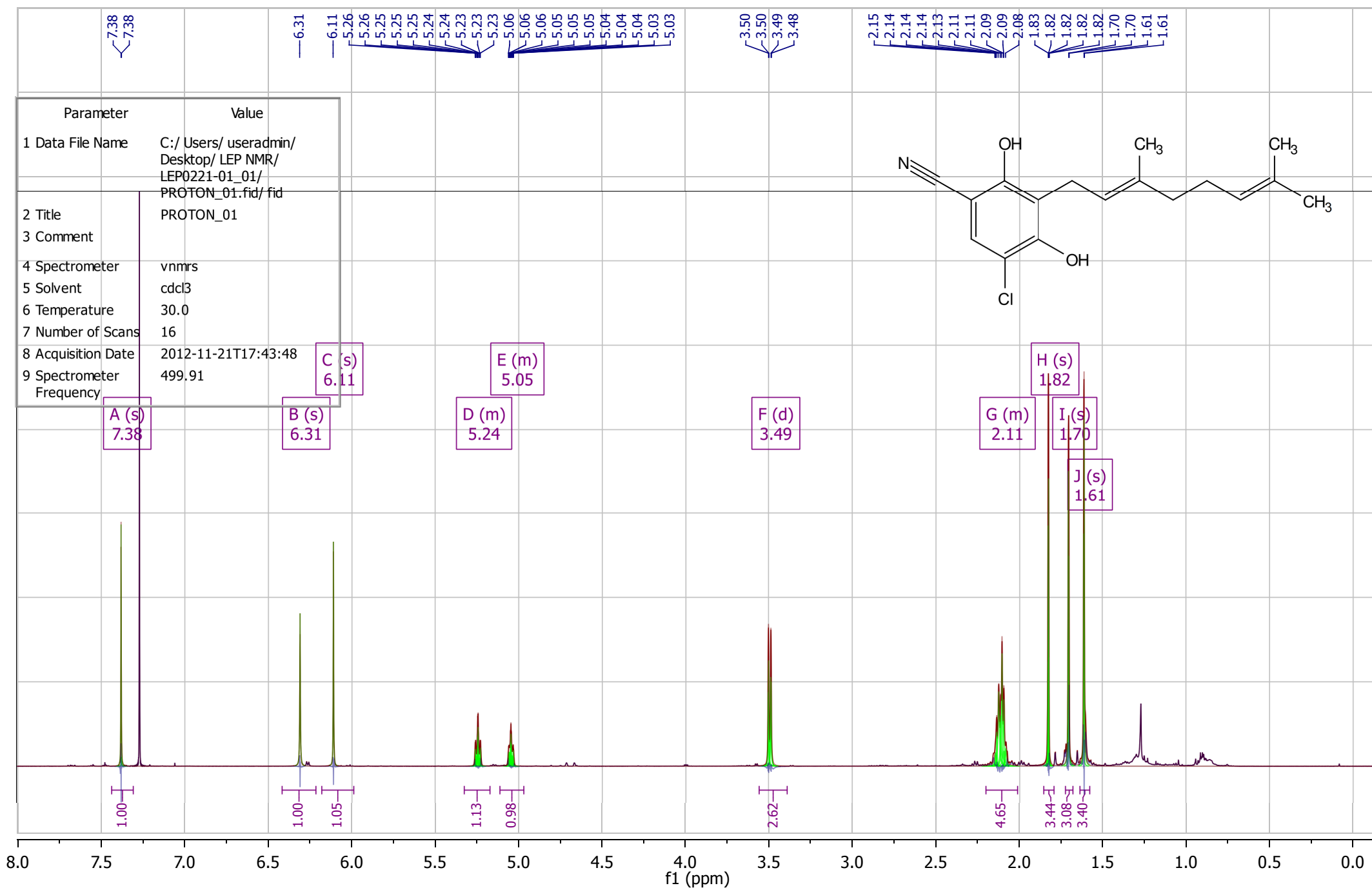

$^1\text{H}$  NMR (500 MHz, Chloroform- $d$ )  $\delta$  7.38 (s, 1H), 6.31 (s, 1H), 6.11 (s, 1H), 5.33 – 5.17 (m, 1H), 5.11 – 4.97 (m, 1H), 3.49 (d,  $J$  = 7.4 Hz, 3H), 2.20 – 2.01 (m, 4H), 1.82 (s, 3H), 1.70 (s, 3H), 1.61 (s, 3H).

Supplement: LEP-0221-01 [file mmc7.pdf]
